# Cell Surface Engineering Tools for Programming Living Assemblies

**DOI:** 10.1002/advs.202304040

**Published:** 2023-10-12

**Authors:** José Almeida‐Pinto, Matilde R. Lagarto, Pedro Lavrador, João F. Mano, Vítor M. Gaspar

**Affiliations:** ^1^ Department of Chemistry CICECO‐Aveiro Institute of Materials University of Aveiro Campus Universitário de Santiago Aveiro 3810‐193 Portugal

**Keywords:** cell assemblies, cell surface engineering, living materials, tissue engineering

## Abstract

Breakthroughs in precision cell surface engineering tools are supporting the rapid development of programmable living assemblies with valuable features for tackling complex biological problems. Herein, the authors overview the most recent technological advances in chemically‐ and biologically‐driven toolboxes for engineering mammalian cell surfaces and triggering their assembly into living architectures. A particular focus is given to surface engineering technologies for enabling biomimetic cell–cell social interactions and multicellular cell‐sorting events. Further advancements in cell surface modification technologies may expand the currently available bioengineering toolset and unlock a new generation of personalized cell therapeutics with clinically relevant biofunctionalities. The combination of state‐of‐the‐art cell surface modifications with advanced biofabrication technologies is envisioned to contribute toward generating living materials with increasing tissue/organ‐mimetic bioactivities and therapeutic potential.

## Graphical abstract

Cell surface engineering can be explored for generating multicellular living assemblies with user‐defined designs and biological programmability. This review provides a comprehensive overview of currently available toolboxes, as well as presents a critical discussion on the most recent advances and exploitable paths to open potential applications of surface functionalized cells in biotechnology and healthcare.

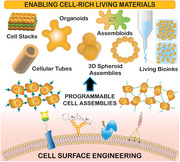

## 1 Introduction

The mammalian cell surface is a complex frontier that plays a central role in modifying cellular behavior and physiological processes.^[^ ^1^ ^]^ Being comprised of a plethora of lipids, proteins, and glycans that carry surface ligands and receptors, the cell surface receives and responds to various stimuli throughout life, operating as a master regulator of major biological processes including i) cell‐environment communication; ii) cell–cell communication, and, iii) intracellular processes activation.^[^ ^2^, ^3^, ^4^, ^5^, ^6^, ^7^, ^8^ ^]^

Cell surface engineering is emerging as a powerful strategy to manipulate and control cell interactions and phenotypes for various biomedical applications. The field has witnessed significant advances in recent years, with a vast array of techniques for engineering the cell surface and artificially tuning intrinsic functions currently available. The cell surface can be modified with different functional molecules such as bioorthogonal chemical groups,^[^ ^9^, ^10^ ^]^ synthetic and natural polymers,^[^ ^2^, ^11^, ^12^, ^13^, ^14^ ^]^ nanoparticles,^[^ ^15^, ^16^ ^]^ proteins/peptides,^[^ ^17^, ^18^ ^]^ or nucleic acids,^[^ ^8^, ^19^, ^20^ ^]^ to enhance their properties and enable specific interactions with other cells or biomaterials. Various techniques have been developed to modify cell surfaces, including covalent conjugation, electrostatic functionalization, hydrophobic insertion, biomolecular recognition, genetic engineering, enzymatic modification, and metabolic engineering.^[^ ^21^ ^]^ These techniques provide precise control over the type and density of functional molecules on the cell surface, allowing for customized cell interfaces with user‐defined properties. Engineering cell surfaces through these approaches offer excellent potential for drug delivery,^[^ ^16^, ^22^ ^]^ bioimaging,^[^ ^23^ ^]^ targeted cell‐based therapies,^[^ ^24^ ^]^ transfusion,^[^ ^11^ ^]^ cell behavior manipulation,^[^ ^25^ ^]^ and tissue engineering applications.^[^ ^26^ ^]^

On this focus, researchers have been incorporating bioactive peptides or proteins on the cell surface to promote specific cell adhesion or modulate cell signaling pathways.^[^ ^27^ ^]^ This approach has been used to create cell‐based biosensors, where cells are engineered with specific surface receptors that can detect and respond to target molecules.^[^ ^28^ ^]^ In addition to sensing applications, cell surface engineering plays a crucial role in tissue engineering and regenerative medicine (TERM), especially in the development of programmable cell assemblies and living materials. Relying on current toolboxes, researchers are actively exploring the use of functionalized cells as unitary building blocks for generating higher‐order bioarchitectures.^[^ ^21^ ^]^ By introducing complementary molecular interactions on different cell surfaces, researchers can orchestrate the self‐assembly of cells into complex structures.^[^ ^26^, ^29^, ^30^ ^]^ This approach has been used to create multicellular architectures, such as cell sheets, clusters, or tissue‐like architectures, with controlled spatial organization.^[^ ^31^, ^32^, ^33^ ^]^ Furthermore, integrating genetic circuits into cell surfaces allows for the dynamic control of cell–cell interactions, enabling the development of artificial cellular systems with advanced functionalities.^[^ ^34^ ^]^ Overall, engineered cell assemblies can be tuned according to the type of surface modification and assembly mode.

Gathering on this potential, herein we aim to highlight innovative cell surface engineering technologies and explore the recent progress of these approaches in programming cellular interactions that in the future will be used for generating living assemblies exhibiting tailorable physiomimetic functionalities according to their envisioned biomedical application. Particularly, chemically‐ and biologically‐driven modifications of cell surface elements (i.e., native, and artificially installed) are addressed and critically discussed, considering their applicability and potential advances in the future. Alongside, the use of such strategies toward fabricating bottom‐up engineered cell assemblies with bioadaptive features is presented, considering their potential to impact major areas of knowledge including fundamental and developmental biology, tissue repair, and disease modeling, among others.^[^ ^35^, ^36^, ^37^ ^]^ Last, a critical analysis regarding the current and foreseeable advances of cell surface engineering for programming living materials is provided, with a particular emphasis on the infancy of the field and the potentially valuable combination with biofabrication approaches to design macro‐scale lab‐grown tissue/organs.

## 2 Engineering Toolsets for Modifying the Cell Surface

**Figure 1:**
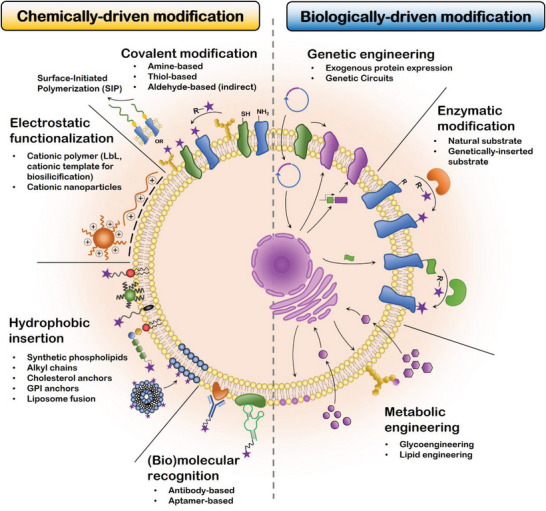
Schematics of currently available cell surface engineering toolboxes for engineering cell surfaces, including chemically‐ and biologically‐driven approaches. Chemically‐driven approaches take advantage of the naturally occurring landscape of chemical and biophysical features of the cell surface. Biologically‐driven approaches use intrinsic biological machinery or bioactive mediators (i.e., enzymes) to install added functionalities in living cells. Such toolsets enable the processing of cells into randomly aggregated or spatiotemporally programmed living assemblies.

The surface of mammalian cells stands as an intricate and refined structure that acts to strictly control cellular behavior and physiological processes in natural biosystems.^[^ ^38^ ^]^ The diverse biomolecular landscape found at the cell surface encompasses numerous reactive hotspots that can be manipulated by using diverse chemical, physical, and biological technologies. Such approaches can ultimately be used to redefine cell surface biological activity and/or add new cell processing possibilities (**Figure** **1** **)**.^[^ ^39^ ^]^ From the plethora of elements that comprise the cellular membrane, the existing reactive chemical groups available in proteins and carbohydrate residues, as well as the intrinsic negative charge and hydrophobicity of the plasma membrane itself constitute natural binding hotspots that researchers can explore to engineer the cell surface and to modulate bioactivity from the outside of the cell, that is, without manipulating intracellular processes. On the other hand, some approaches have explored the intracellular biosynthetic machinery and natural metabolic processes to install new natural or unnatural chemical moieties/biomolecules to further attain a more specific modification of target elements on the cell surface, including the cell membrane or glycocalyx.^[^ ^40^ ^]^ To precisely exploit the natural biochemical mechanisms and machinery of living cells, researchers have been actively exploring genetic and metabolic engineering toolboxes. This has allowed the manipulation of the display of desired moieties for augmenting cell surface processability and, ultimately, for programming the assembly of higher‐order multicellular architectures. Alongside, important biological elements, such as enzymes, have also been exogenously applied to selectively modify certain native or non‐natively tagged elements on cell surfaces, allowing a selective manipulation of the displayed biomolecular landscape. Considering the bioengineering potential and diversity of these toolsets, here we have classified the different cell surface engineering technologies into two major classes: i) chemically‐ and ii) biologically‐driven modifications, which will be the focus of discussion in the following sections.

### 2.1 Chemically‐Driven Cell Surface Engineering

#### 2.1.1 Covalent‐Based Functionalization

The cell surface is decorated with a wide range of naturally existing functional chemical groups in biomolecules, among which amines, thiols, and carboxylic acids are readily available for further modification via precise chemical reactions.^[^ ^4^, ^5^ ^]^ By exploring the potential of these naturally available groups, chemical covalent modification is considered a simple and straightforward method for cell surface modification, as it enables a direct attachment of different biomolecules/biomaterials with complementary functional groups without requiring the involvement of the intracellular machinery or genetic approaches.^[^ ^4^, ^41^ ^]^ Amine (‐NH_2_) and thiol groups (‐SH), displayed at lysine and cysteine amino acid side chains, are well‐known attachment points, allowing direct surface modification without requiring chemical or genetic preconditioning of the cell.^[^ ^5^ ^]^

Amine‐based modifications are commonly performed by reacting native amines and activated carboxylic groups present in pre‐activated exogenous functional moieties displaying: i) cyanuric chloride, ii) *N*‐hydroxysuccinimide (NHS) esters, and/or iii) aldehyde groups, among others.^[^ ^3^, ^42^ ^]^ Pre‐activation of exogenous molecules is of utmost importance, since without the activation of carboxylic groups, the binding with native primary amines could be compromised by the deprotonation of carboxylic acids instead of the formation of stable amide bonds.^[^ ^30^ ^]^ Cyanuric chloride‐modified molecules have been used in amine‐based cell surface engineering due to the selective reaction with primary amines.^[^ ^43^ ^]^ Despite presenting a relatively high functionalization efficiency and rapid conjugation time, the lack of cytocompatibility caused by harmful side products limits the applicability of this strategy. Similarly, using NHS‐esters for conjugation is an appealing approach for selectively modifying cell surface primary amines.^[^ ^44^ ^]^ Compared to cyanuric chloride, NHS‐esters present higher cytocompatibility and lack harmful byproducts, resulting in NHS‐esters being considered the best strategy for cell surface amine modification.^[^ ^6^ ^]^ Beyond both approaches, aldehyde‐containing molecules can also be used to modify cell surface amines through azaelectrocyclization by reacting with unsaturated ester aldehyde moieties, or through the formation of a Schiff base, that can be further reduced by sodium cyanoborohydride to promote the formation of more stable covalent bonds.^[^ ^4^, ^42^, ^45^, ^46^ ^]^

Thiols are another abundant group present in the cell surface, these can be found in either oxidized (i.e., disulfide bridges) or reduced form (i.e., free thiols), being its biological balance dictated by the surrounding redox microenvironment.^[^ ^4^ ^]^ Due to their nucleophilicity, free thiols represent a valuable source to introduce chemical modifications in the cell surface.^[^ ^41^, ^47^ ^]^ However, the majority of surface thiols are naturally found in their oxidized form which may limit their availability for cell surface engineering approaches.^[^ ^2^ ^]^ Even so, by simply altering the reaction conditions via mild reduction agents (i.e., TCEP),^[^ ^15^, ^48^ ^]^ cell surface disulfide bonds can be converted into free thiols which are reactive and readily available to be modified, albeit generally in a non‐bioorthogonal mode in comparison to other more selective click‐chemistry strategies.^[^ ^2^, ^4^ ^]^ The most common approaches for engineering the cell surface using free thiols are through conjugation with elements that display: i) maleimides or ii) pyridyldithiol.^[^ ^41^, ^49^, ^50^ ^]^ Maleimide‐containing molecules have been the most widely used complementary conjugation partners, ensuring chemoselectivity and the formation of stable and irreversible thioether bridges, through Michael‐type addition reaction.^[^ ^4^, ^5^, ^6^, ^41^ ^]^ If a degradable binding is intended, pyridyldithiol‐containing molecules are a common option, reacting with thiols through the formation of reversible disulfide bonds.^[^ ^50^, ^51^ ^]^ Besides the well‐reported amine‐ and thiol‐based strategies, *cis*‐diol units can also be naturally found in glycoproteins along the cell surface, especially in sialic acid and galactose residues.^[^ ^38^ ^]^ These functional groups can be made available for surface modification by resorting to dynamic covalent chemistry (DCC) through reaction with boronic acids, yielding boronate‐ester linkages, allowing researchers to dynamically control and revert the conjugation by tuning the levels of free glucose in the medium.^[^ ^52^ ^]^ This surface modification toolset is particularly valuable if the fabrication of dynamic cell assemblies is envisaged.

Besides the naturally available functional moieties found in the cell surface, the native chemical toolbox can also be amplified by introducing unnatural groups without resorting to the cell machinery, particularly by oxidizing sialic acids residues using sodium periodate to yield aldehyde groups on glyco‐elements of the cell surface, a method so termed—periodate oxidation.^[^ ^3^, ^4^, ^53^ ^]^ Unlike amine‐ and thiol‐based modifications, aldehyde‐based modifications require a chemical preconditioning stage to install such moieties. Nonetheless, this unlocks new possibilities for promoting chemical modification of the cell surface through conjugation with amine, aminooxy, and hydrazine groups, installing new functionalities via DCC through the establishment of imine bonds, including Schiff base, oxime, and hydrazone linkages, respectively.^[^ ^3^, ^54^, ^55^, ^56^, ^57^ ^]^ In addition to requiring cell chemical preconditioning, aldehyde‐based approaches could be hindered by uncontrolled reactions between introduced aldehydes and native amines, leading to undesired crosslinking between cell surface elements, which could ultimately originate an inefficient modification.^[^ ^53^ ^]^

In general, these covalent chemical modifications provide a stable and long‐lasting functionalization of the cell surface and can be easily performed due to the natural abundance of diverse chemical groups in the cell surface. However, the applicability of this approach is relatively limited, owing to the lack of targeting specificity and the poor control over the extension of the chemical modification. Such could lead to unwanted physiological alterations in the cell membrane and a loss of surface protein bioactivity, ultimately affecting cell bioactivity.^[^ ^30^, ^50^ ^]^ Some of these drawbacks may be overcome by exploring non‐covalent and biologically‐driven approaches with non‐permanent features, as will be further discussed. Besides chemical functional groups displayed in the cell surface, non‐covalent approaches have also been explored to perform cell surface engineering under mild conditions that do not require modifications to the cells' native components. Non‐covalent approaches take advantage of cell's physical aspects, especially its negatively charged surface and hydrophobic membrane, as well as molecular recognition mechanisms.

#### 2.1.2 Non‐Covalent Surface Engineering Toolsets

##### Electrostatic Functionalization

A combination of different negative elements, including sialic acid residues on glycoproteins, phosphate groups on phospholipids, and carboxyl groups of proteins, confers a negatively charged nature to the cell membrane.^[^ ^39^ ^]^ Since the negatively charged cell membrane is transversal to all mammalian cell types, this approach is highly generic and straightforward. From a bioengineering perspective, such a negative charge renders the cell membrane a potential binding site for positively charged materials/biomolecules to electrostatically interact and adsorb.^[^ ^58^ ^]^ The electrostatic binding modification consists of a simple and inexpensive approach to redefine cell surface with new physical and chemical properties that could further expand the functionalities of single cells, upon the introduction of new functional groups, as well as confer mechanical support and protection against external stress.^[^ ^59^, ^60^ ^]^

For establishing multivalent electrostatic interactions different positively charged materials have been successfully installed onto the cell surface, including diverse positive polymers such as poly(ethylenimine) (PEI), poly‐L‐lysine (PLL), and chitosan, as well as positive nanoparticles.^[^ ^59^, ^61^, ^62^, ^63^ ^]^ Despite the simplicity of surface engineering through electrostatic functionalization, the high charge density produced by the majority of polycations may potentially result in cell damage upon internalization, or disrupt the cell's membrane integrity.^[^ ^41^, ^61^ ^]^ This drawback has been to date attenuated by combining spacers, typically poly(ethylene glycol) (PEG), to curtail the direct contact between the positively charged material and the cell surface, as a strategy for minimizing cytotoxicity. Alternatively, the selection of biocompatible biopolymers for coating mammalian cells has also been explored as a suitable option.^[^ ^50^, ^60^, ^64^ ^]^

Among the different methodologies that have been employed to modify the cell surface, the alternate adsorption of oppositely charged polyelectrolytes, the so‐called layer‐by‐layer (LbL) technique, has been the most widely used.^[^ ^65^ ^]^ This methodology leads to the formation of a thin polyelectrolyte multilayer (PEM) film around the cell surface, forming a physical barrier. Such an approach has been particularly useful in the context of cell therapies/transplantation applications, as the cell coating with nanosized multi‐layers creates a physical barrier that hinders the recognition by the host immune system, thus limiting rejection events.^[^ ^4^, ^5^, ^6^, ^50^ ^]^ In addition, this technology could be used to sequentially install cell layers intercalated with PEM films to fabricate multi‐layered tissue‐like architectures.^[^ ^65^, ^66^, ^67^ ^]^ Although such a strategy was successfully demonstrated, it remains a cumbersome and lengthy process for achieving cell surface functionalization. Alternatively, a simple attachment of positively charged polymers could provide a faster and more convenient way to install new functional moieties onto the cell surface. Such cationic polymers could also be potentially functionalized with chemical moieties or hydrophobic anchors, resulting in a synergistic dual‐modification method with enhanced incorporation efficiency and stability. This could be useful in scenarios where the multivalent electrostatic interactions alone are not strong enough to promote cell aggregation.^[^ ^68^, ^69^ ^]^ Alongside, silicification processes involving the adsorption of silicates into a cationic polymer, previously installed electrostatically on the cell surface, have also been used to promote the functionalization of single cells and generate multicellular assemblies, as will be further highlighted.^[^ ^59^, ^61^, ^70^ ^]^ The widespread applicability of these methodologies is however limited by a lack of cell‐type selectivity, which could be a challenge for applications that aim to explore one‐step, cell‐specific modification in the context of heterotypic living constructs assembly.

##### Hydrophobic Insertion

The mammalian cell membrane is comprised of a substantial fraction of lipidic material, especially a phospholipid bilayer, which creates a boundary between the intra‐ and extracellular environments, as well as other embedded molecules, such as cholesterol.^[^ ^8^, ^41^, ^71^ ^]^ The natural arrangement of these molecules results in a hydrophobic region, which constitutes an attractive spot for cell surface engineering through the insertion of lipophilic/hydrophobic molecules. By exploiting this lipophilic nature, the scientific community has been able to introduce modifications by simply mimicking key aspects of the bilayer, allowing an easy and rapid anchoring of modified molecules with minimal impact on cell viability and bioactivity, when compared to some covalent and electrostatic‐based modifications.^[^ ^41^ ^]^

The hydrophobic insertion of unnatural molecules/moieties in the cell surface has been mainly performed by lipid anchoring upon the covalent linkage of the molecule of interest. Most common lipid anchors include: i) synthetic phospholipids, ii) alkyl chains, and iii) other lipidic molecules (i.e., cholesterol and glycosylphosphatidylinositol (GPI) anchors), which are inserted and immobilized in the hydrophobic region of the existing phospholipid bilayer by the establishment of hydrophobic interactions.^[^ ^72^ ^]^ Due to the abundance of phospholipids in the cell membrane, synthetic phospholipids (i.e., 1,2‐dimyristoyl‐*sn*‐glycero‐3‐phosphoethanolamine)^[^ ^18^ ^]^ have been widely used as hydrophobic moieties for hydrophobic insertion, due to their structural similarity to native phospholipids.^[^ ^41^ ^]^ Alternatively, alkyl chains (i.e., C18 chain)^[^ ^17^ ^]^ could also be covalently linked to molecules of interest, and act as hydrophobic anchors, expanding the hydrophobic insertion toolbox. Yet, when designing such strategies, it is important to consider that the cell membrane architecture is highly dynamic and complex. Thus, a careful selection and design of the hydrophobic anchor is critical to achieve the ideal anchoring efficiency, which is strongly related to chain hydrophobicity and structural similarity to phospholipids.^[^ ^5^, ^73^ ^]^ In this approach, hydrophobicity is dictated by carbon chain length, as well as chain saturation, where long and saturated lipidic chains tend to achieve relatively higher anchoring efficiency and more stable integration into the mammalian cell membrane.^[^ ^5^, ^73^ ^]^ In terms of structural similarity, synthetic lipid‐ and dialkyl‐conjugated molecules tend to achieve, in general, a higher anchoring efficiency and homogeneous display in the cell surface, as they more closely resemble a two‐tail lipid.^[^ ^73^, ^74^ ^]^ Similar to alkyl chain conjugation, cholesterol has also been exploited as a hydrophobic anchor to non‐covalently attach different materials to the cell surface.^[^ ^75^, ^76^ ^]^ Cholesterol‐functionalized materials usually exhibit considerable anchoring efficiency.^[^ ^74^ ^]^ However, due to living cells' natural membrane dynamics, a rapid cholesterol exchange could occur, resulting in a time‐limited modification.^[^ ^74^ ^]^ Such aspect, combined with the difficulty in the chemical preparation of the cholesterol conjugates, renders this approach rather challenging and requires extensive optimization for achieving optimal cell surface engineering.^[^ ^41^ ^]^ Gathering on the rapid advances in the DNA nanotechnology field, self‐assembled DNA structures bearing multiple cholesterol anchors have been designed to overcome the abovementioned limitations. The resulting cholesterol‐DNA structures display improved anchoring stability and a longer‐lasting cell surface modification.^[^ ^77^ ^]^ Alternatively, glycosylphosphatidylinositol (GPI) anchors are also emerging as another major technology to install diverse moieties on the cell surface. This simple methodology relies mostly on a mechanism found in biological processes. During physiological processes, GPIs are post‐translationally inserted in a plethora of cell‐surface proteins to anchor them into the cell surface.^[^ ^39^, ^78^ ^]^ By mimicking this concept, not only proteins but also other functional moieties can be conjugated with GPI anchors, commonly through chemical conjugation. Besides non‐genetic modification of the GPI anchors, some approaches have used genetic engineering tools to recombinantly install peptides/proteins directly and, after purification, to install them into cell surface—fusion protein technology—that will be further discussed below.^[^ ^79^ ^]^

A common problem with hydrophobic insertion through lipid anchoring is the possibility of functional moieties internalization due to the hydrophobic character of the conjugates.^[^ ^73^ ^]^ To address this issue, hydrophilic polymers are commonly applied as a spacer between hydrophobic and functional moieties, as a strategy to improve molecular immobilization at the cell surface and avoid internalization. PEG has been, to date, the polymer of choice to enhance the hydrophilic character of the conjugate, with various recent reports evidencing its potential to reduce internalization and improve cell surface functionalization efficiency.^[^ ^71^, ^73^, ^80^ ^]^ In addition, the PEG spacer enhances the stability of the conjugate by attenuating the steric hindrance effect that can occur between surface molecules and the installed functional moieties.^[^ ^81^ ^]^ However, in these strategies, the PEG molecular weight should be carefully selected and optimized to block internalization without interfering with receptor/ligand binding or inhibiting the desired lipid anchoring.^[^ ^73^ ^]^

In addition to lipid anchoring, liposome fusion has been exploited as an alternative approach to installing non‐natural functional moieties on the cell surface by relying on hydrophobic interactions and cell membrane fusion. By mimicking the spontaneous cell membrane fusion processes, biofunctionalized fusogenic liposomes containing unique phospholipids can interact andefficiently fuse  into the cell, allowing the incorporation of large sections of the phospholipid content into the cell membrane.^[^ ^5^, ^26^, ^30^, ^50^ ^]^ A unique feature of this methodology is the possibility of modifying both the inner and outer membrane, which could be an interesting approach to studying cellular behavior or intracellular signaling while manipulating the outer membrane.^[^ ^82^ ^]^

Although hydrophobic insertion generally presents cell‐type independence, the intrinsic dynamics of the membrane of different types of cells could lead to discrepancies in insertion efficiency between different hydrophobic anchors, as well as the whole functional conjugate. Thus, during design stages, adequate screening and optimization of lipid anchors and liposomes are critical to achieve optimal insertion efficiency and balanced cell bioactivity.^[^ ^26^, ^73^ ^]^ The attractivity of this technique centers around its speed and ease of carrying out relatively harmless cell modifications, generally exhibiting higher cytocompatibility when compared to some conventional covalent modifications and electrostatic binding approaches.^[^ ^5^ ^]^ However, as the mechanism of hydrophobic insertion is based on non‐covalent interactions, the inserted moieties usually suffer from rapid and passive dissociation from the cell surface.^[^ ^83^ ^]^ In addition to passive dissociation, the performance of hydrophobic insertion could also be hindered by intrinsic membrane events, specifically, the activity of flippases, generally responsible for causing a “side‐switch” of the non‐native molecules anchored in the outer leaflet to the inner leaflet. Such could lead to potential undesirable effects on cell behavior and viability, as well as a decrease in functional moieties availability.^[^ ^5^ ^]^ Carefully addressing these parameters according to the cell type and the required functionalization lifetime is key to generating highly tunable living cell assemblies at different length scales.

##### Biomolecular Recognition

Relying on the non‐covalent installation of functional moieties, surface engineering through biomolecular recognition constitutes another attractive tool that has been recently explored for programming the cell surface with new features that prove beneficial for generating living cell assemblies. Up‐to‐date, biomolecular recognition approaches have mostly exploited the high affinity of antigen‐antibody interactions or aptamer‐target affinity, among others.^[^ ^15^ ^]^

Antibody‐based modification relies on the natural recognition between an antibody and a specific antigen on the cell surface. Due to antibodies’ relatively low dissociation rate (e.g., on the order of 10^−5^ s^−1^) and high specificity, they can be used to selectively install new functionalities on the cell surface.^[^ ^84^ ^]^ Alternatively, aptamers represent valuable molecular recognition ligands for programming cell–cell and cell–material interactions toward creating multicellular living assemblies. Aptamers generally comprise a well‐defined sequence of single‐stranded DNA or RNA oligonucleotides, that directly interact with a specific target (i.e., extracellular domains (ECDs) of membrane proteins, carbohydrates, etc.).^[^ ^85^, ^86^ ^]^ By adopting a specific secondary or tertiary structure, these biomolecules have relatively high binding affinity toward their targets.^[^ ^8^, ^87^ ^]^ Interestingly, aptamers are also often associated with antibody‐like activities, as they are chemically synthesized and operate in similar mechanisms of ligand recognition, where mutual matching of spatial conformations with their targets is required.^[^ ^88^ ^]^ The selection of aptamer candidates with the highest specificity and binding affinity to the intended biomolecular target can be identified by using the systematic evolution of ligands by exponential enrichment (SELEX) technique.^[^ ^71^ ^]^ Instead of targeting a specific cell surface receptor, cell‐type‐specific aptamers can be selected by a variant of the SELEX technique, cell‐SELEX,^[^ ^89^ ^]^ which allows the screening of specific cell‐directed aptamers without requiring prior knowledge of the target signature.^[^ ^19^, ^88^, ^89^ ^]^ Such technique provides immense possibilities for bioengineering cell‐specific interactions up to the level of surface‐expressed proteins tailored to different cell states (i.e., homeostasis, inflammation, cancer, etc.).^[^ ^88^, ^90^ ^]^ Aptamers are relatively easy to synthesize, purify and post‐process, exhibit a potentially lower immunogenicity, and relatively long‐term stability, as well as a smaller size, which reduces the risk of causing undesired structural perturbations in biomolecules’ structure.^[^ ^88^, ^90^, ^91^ ^]^

Generally, antibody‐ and aptamer‐based approaches for cell surface engineering require covalent modification with functional chemical groups. In antibody engineering approaches, the covalent attachment of functional moieties in these units is conventionally performed through their lysine or cysteine residues, which commonly leads to heterogeneous products, limiting their applicability.^[^ ^92^ ^]^ More recently, efforts have been made toward producing homogeneously modified antibodies through the introduction of bioorthogonal moieties that can be further used to attach the functional moieties through bioorthogonal conjugation reactions, showing superior results in comparison to their more heterogeneous counterparts.^[^ ^92^, ^93^ ^]^ However, such processes often result in undesirable effects on antibody folding and stability, in addition to being considerably more costly.^[^ ^92^, ^93^ ^]^ On the other hand, the chemical modification of aptamers is more flexible, allowing a site‐specific introduction of functional chemical groups with stoichiometric accuracy, having, in general, greater flexibility in comparison to antibody modification.^[^ ^88^ ^]^ Besides the introduction of reactive chemical groups, aptamers are usually chemically modified to enhance their stability and resistance against nuclease‐mediated degradation.^[^ ^71^, ^88^, ^94^, ^95^ ^]^ Furthermore, in the case of DNA conjugation, the chemical modification of aptamers is avoided, as they can be designed with the desired DNA tail, rising from the aptamer nucleic acid body.^[^ ^71^ ^]^ Based on the advances in antibody engineering, different alternatives to conventional monoclonal antibodies (mAbs) can be applied in the cell surface engineering field, including antibody fragments (i.e., single‐chain variable fragments, nanobodies, antigen‐binding fragments, etc.), and bispecific antibodies. These can ultimately provide multiple advantages over standard mAbs and prompt the development of innovative living assemblies for TERM applications.^[^ ^96^, ^97^, ^98^ ^]^

Besides antibody‐antigen and aptamer‐target cell surface engineering, targeting peptides have also been explored as a biocompatible platform based on their ability to target specific receptors on the cell surface. Despite having a relatively lower binding affinity compared to antibodies, targeting peptides are a smaller‐sized option with relevant advantages, including low immunogenicity, increased penetration, and high availability, rendering them interesting alternatives for surface functionalization.^[^ ^99^, ^100^ ^]^

The use of vitamin‐protein combinations has also been a widely explored biomolecular recognition approach for cell surface engineering in recent years. In this context, biotinylation is one of the most valuable approaches for cell surface engineering, allowing researchers to insert different modifications by exploring the strongest known natural molecular recognition, which is established between biotin and avidin, or its analogs streptavidin and NeutrAvidin.^[^ ^15^ ^]^ Due to its high affinity and strong resistance to degradation, the biotin‐avidin interaction has been widely used in cell surface engineering.^[^ ^101^ ^]^ However, this special tool is only feasible after a previous installation of biotin molecules onto the cell surface, generally through chemically‐ and/or biologically‐driven modifications, enabling a posterior conjugation with avidin‐functionalized materials.^[^ ^102^, ^103^ ^]^ Considering this, the biotinylation process and associated biotin display are herein regarded mainly as an indirect modality that can be used to install new functionalities on the cell surface. Similarly, cyclodextrins (CDs) and cucurbit[7]uril (CB[7]) molecules are also considered bridging functional moieties, relying on an engineering method to be installed onto cell surface, so that they can further interact with their guest pairs, including (e.g., azobenzene, adamantane (Ada), etc.), through host‐guest interactions and establish supramolecular inclusion complexes.^[^ ^104^, ^105^ ^]^

Even though molecular recognition tools show great potential for precision cell surface engineering, especially considering their target specificity and binding affinity, the dependency on naturally occurring binding sites could still be a limiting factor for the reproducibility and anchoring efficiency of this approach.^[^ ^8^ ^]^ Exploring other tools that are not limited to the natural existence of available chemical groups on the cell surface is also required for the field to advance, as these can provide an added degree of programmability in the moieties that can be installed and exploited. In this framework, the rapid evolution of bioengineering techniques for manipulating living cells is creating new opportunities to modulate cell surface composition, as will be discussed in the following sections.

### 2.2 Biologically‐Driven Cell Surface Modification

#### 2.2.1 Genetic Engineering

Genetic engineering is a well‐established and versatile tool to manipulate the inclusion of specific proteins on the cell surface in a fully biologically‐driven mode. This approach leverages the biosynthetic processing of exogenous genetic material inserted into the cell to modulate the expression of desired proteins on the cell surface. The use of genetic engineering methodologies to deliver genetic cargo (i.e., plasmid‐ and minicircle DNA (pDNA and mcDNA), messenger RNA (mRNA), etc.), and/or non‐coding regulatory elements (i.e., micro RNA (miRNA) and small interfering RNA (siRNA)), is one of the most established and attractive applications for generating cell surface engineered therapeutics, both at a preclinical and clinical level.^[^ ^106^, ^107^, ^108^, ^109^, ^110^ ^]^ For example, these technologies are being explored to genetically engineer T‐cells with chimeric antigen receptor (CAR) upon incorporation of user‐designed exogenous material, allowing cells to express an artificial cell surface receptor that recognizes a specific cancer cell antigen.^[^ ^111^ ^]^ Up‐to‐date, different gene delivery vehicles have been used to transport and deliver genetic material to the intracellular milieu, including viral vectors (i.e., lentivirus, gamma‐retrovirus, adenovirus, adeno‐associated virus, herpes simplex virus)^[^ ^112^, ^113^ ^]^ and non‐viral vectors (i.e., cell‐derived vesicles, nanoparticles, etc.).^[^ ^114^, ^115^ ^]^ Nucleic acid delivery efficiency is key for genetic engineering success, as the desired protein expression strongly depends on the internalization of the exogenous genetic material.^[^ ^50^ ^]^ Considering this, viral vectors have been one of the most employed delivery systems for genetic engineering due to their considerable transduction efficiency, resulting in a stable or transient expression depending on the selected viral vector.^[^ ^109^ ^]^ However, the use of viral vectors constitutes a major concern owing to a higher risk of insertional mutagenesis, as well as a higher probability of triggering immunogenic responses, potentially compromising safety in specific clinical applications.^[^ ^72^, ^73^ ^]^ To attenuate some concerns about the use of viral vectors, the search for non‐viral alternatives has recently intensified. In general, such vectors can be used to promote the delivery of stable or transient expression cassettes and, generally, vary in transfection efficiency.^[^ ^109^ ^]^ For example, nanoparticles (e.g., lipid, polymeric, inorganic, bio‐derived vectors such as exosomes, etc.) represent highly customizable platforms that can be tuned with target delivery features.^[^ ^109^ ^]^ Alternatively, electroporation has been widely used to achieve a transient expression with a suitable transfection efficiency, but the resulting toxicity generally caused by the high voltages applied could hinder its broad application. Compared to the previously presented methods, non‐viral nanosized delivery systems can be generally engineered to show relatively low toxicity, providing a great opportunity for in vivo applications of these technologies. Despite their relatively low transfection efficiency, current efforts in the materials engineering field have resulted in significant progress, especially in the development of improved cell‐based therapies and new vaccines, namely those based on liposomal platforms. Additionally, increasing pieces of evidence indicate that some cell types are naturally less susceptible to transfection, especially stem cells and some types of endothelial cells (e.g., vascular cells, fibroblasts, etc.), so major improvements are required for this strategy to be considered as a “one‐fits‐all” approach.^[^ ^50^, ^78^ ^]^

In line with envisioned advances, the use of genome editing technologies (i.e., zinc finger nucleases, transcription activator‐like effector nucleases, clustered regularly interspaced short palindromic repeats/Cas9 or Prime editing) provides powerful editing tools for precise gene manipulation, revealing great potential to revolutionize the cell surface engineering field and cell‐based therapies.^[^ ^107^, ^116^, ^117^, ^118^ ^]^

Many of the aforementioned technologies have been used in the field of synthetic biology to include genetic circuits and light‐responsive proteins (i.e., optogenetic approaches) that allow the generation of programmable living cell assemblies whose interactions can be precisely controlled over time. Such technologies also have the potential to provide deeper insight into the spatiotemporal self‐organization of multicellular architectures.^[^ ^119^, ^120^ ^]^

Inside the toolbox of synthetic biology, the engineering of synthetic genetic circuits is a highly attractive and robust approach for inducing morphological changes through the incorporation of cell–cell signaling networks.^[^ ^27^ ^]^ From the plethora of programmable genetic circuits, synthetic Notch (synNotch) receptor installation has been the most widely used for cell surface functionalization, enabling precise control over cell–cell interactions through juxtracrine signaling.^[^ ^121^ ^]^ Based on the heterologous modification of the extra‐ and intracellular domains of transmembrane Notch proteins, the synNotch receptor represents a highly customizable molecular recognition element, responding to certain inputs, and culminating in the activation and expression of desired genes. The use of multiple synNotch networks has been recently explored for developing synergistic cell–cell pathways and inducing new regulatory cascades between cells within living cellular assemblies.^[^ ^34^ ^]^ Such toolbox will be further discussed in light of the possibilities it opens for engineering next‐generation living materials fabrication. Besides synNotch circuit engineering, other signaling circuits can be explored, including G‐protein‐coupled receptor‐based circuits (i.e., Tango, ChaCha), modular extracellular sensor architecture (MESA), and generalized extracellular molecule sensors (GEMS).^[^ ^122^, ^123^, ^124^, ^125^ ^]^ These have been particularly underexplored for programming microenvironment‐responsive living assemblies, and major advances in this direction are envisioned in the upcoming years. Yet, it is relevant to discuss that such genetic circuits present a lower degree of programmability due to ECDs restrictions, where the activation is limited to natural receptor recognition (e.g., Tango and MESA toolsets) or presents a limited number of downstream pathways that could be activated (e.g., GEMS‐based toolsets), thus limiting their versatility and widespread applicability.^[^ ^121^ ^]^ Nonetheless, hybrid constructs combining juxtracrine and paracrine signaling events are envisioned to unlock the fabrication of living materials with programmable sensing/assembling capabilities, potentially combining both membrane‐bound and soluble factor detection as recognition inputs, with customizable activation outputs.

Adding to this toolbox, optogenetics‐based approaches constitute yet another interesting and relatively simpler methodology to control cell–cell interactions through genetically‐induced expression of light‐switchable proteins installed onto the cell surface.^[^ ^121^ ^]^ Upon light irradiation, the expressed proteins tend to dimerize, promoting homo‐ and/or heterophilic interactions between different cell populations. Moreover, due to the differences found in distinct protein‐protein pairs, different dynamics and wavelengths may produce programmable outputs, which can be tuned to modulate the dynamics of cell assembly processes in an on‐demand mode.^[^ ^126^ ^]^ Besides such orthogonal engineering of cell adhesion pairs, the same rationale can also be exploited to modulate cell behavior through native adhesion molecules (i.e., integrin‐mediated cell adhesion), enabling dynamic control over native cell adhesions.^[^ ^127^ ^]^ When rationally designed, optogenetic approaches are powerful tools for establishing dynamic and reversible cellular assemblies in a non‐invasive mode and with higher spatiotemporal resolution, as will be highlighted.^[^ ^119^, ^120^ ^]^

The introduction of non‐native proteins by fusion protein methods is another interesting alternative. In a similar concept to that of hydrophobic insertion, the fusion protein approach consists of an exogenous genetic modification of a desired protein to recombinantly express a membrane anchor, typically a GPI molecule, mimicking the natural GPI anchoring process and consecutively enable surface protein engineering.^[^ ^79^ ^]^ By modifying the genetic sequence of naturally GPI‐anchored proteins, a GPI anchor can be directly incorporated in the desired proteins, thus bypassing the additional conjugation step for chemically interlinking the hydrophobic anchor as observed in hydrophobic insertion methods.^[^ ^5^, ^78^ ^]^ Resorting to this method, the cell surface can be modified with diverse proteins, either simultaneously or sequentially, with precise control over the molar amount of displayed proteins.^[^ ^79^ ^]^ Yet, fusion proteins generally require extensive purification steps before delivery to cells. On the other hand, fusion proteins can also be generated in situ, avoiding complicated and time‐consuming purification processes, while providing interesting platforms to be explored for TERM.^[^ ^128^ ^]^ In addition, in both direct genetic modification and fusion protein approaches, the resulting proteins may show compromised function due to steric hindrances.^[^ ^5^ ^]^

Although conventional genetic engineering methods have proven to be robust means for surface modification, they are somewhat limited to genetically encoded molecules, hindering the modification of the cell surface with unnatural functional biomolecules. In this regard, the genetic code expansion technique has recently been able to circumvent such limitations, allowing the insertion of unnatural functional residues on biomolecules. The Genetic Code Expansion method relies on a completely different idea from the previous genetic approaches, enabling the site‐specific modification of a protein of interest with unique non‐canonical amino acids (NCAA) by genetically remodeling the intrinsic cell translation machinery. Introducing NCAA to the natural amino acid repertoire adds a plethora of new functionalities, breaking the functional limits imposed by the typical 20 amino acids “code” found in most species.^[^ ^129^ ^]^ From a set of possible 64 codons, three of them are blank codons, which do not correspond to any of the 20 canonical amino acids, thus representing potential sites to introduce an NCAA, ultimately expanding the natural genetic code. These, stop codons, UAG (amber), UAA (ochre), and UGA (opal), can be decoded by an inserted orthogonal tRNA.^[^ ^130^ ^]^ To achieve this, a new pair of distinct aminoacyl‐tRNA synthetase (aaRS)/tRNA, that must not react toward endogenous aaRS/tRNA pairs and/or natural amino acids, needs to be expressed.^[^ ^131^ ^]^ During the translation of a modified sequence from a protein of interest, the specific aminoacyl‐tRNA synthetase (aaRS) loads the corresponding distinct tRNA with the desired NCAA, which then decodes the stop codon in a specific location to allow a site‐specific insertion of the NCAA on the desired protein.^[^ ^131^ ^]^ Up‐to‐date, a broad range of non‐canonical amino acids has been successfully incorporated into mammalian cell proteins in a site‐specific manner.^[^ ^129^, ^132^ ^]^ Owing to its minimal occurrence in nature, the amber stop codon has been widely implemented for genetic code expansion. Genetic technologies have also been applied to expand the blank codon repertoire for this technique, especially by improving the capability of tRNAs to recognize and bind to other codons, such as four‐nucleotide codons. In terms of aaRS/tRNA pairs, pyrrolysine aaRS/tRNA pair (PylRS/tRNA^Pyl^) from Archaea *Methanosarcina mazei* and *Methanosarcina barkeri*, and tyrosine, leucine, and tryptophan aaRS/tRNA pairs (TyrRS/tRNA^Tyr^, LeuRS/tRNA^Leu^, and TrpR/tRNA^Trp^) from *Escherichia coli*, are the focus of research in mammalian genetic code expansion method.^[^ ^131^, ^133^ ^]^ Future advancements in their use for engineering cell–rich assemblies with self‐sorting capabilities are envisioned.

#### 2.2.2 Enzyme‐Mediated Cell Surface Functionalization

Due to the inherent site‐specificity and high conversion rates of enzymes, they represent a powerful biological tool for remodeling proteins and glycans displayed at the cell surface. To date, these have been mainly used to enable a highly selective modification of naturally available binding sites in the cell's membrane, under relatively mild conditions that uphold cell viability and biofunctionality.^[^ ^134^ ^]^ Exogenous enzymes catalyze specific enzymatic reactions depending on the presence of specific substrates, allowing in situ modification with functional moieties. In enzyme‐mediated surface modification, enzymes can recognize and transform naturally present substrates or genetically inserted substrates.^[^ ^3^, ^135^ ^]^ A variety of enzymes, including oxidases, transferases, ligases, peptidases, and lipases, have been leveraged to post‐translationally modify a desired set of proteins or glycans.

Enzymatic‐mediated remodeling promotes the modification of naturally displayed proteins and glycans, allowing the conversion of naturally available chemical groups/biomolecular sequences into unnatural functional moieties.^[^ ^3^ ^]^ Using these biomolecular entities as chemical operators, aldehydes can be introduced onto cell surfaces in a fully biologically‐driven mode. Particularly, galactose oxidase recognizes the naturally presented galactose or *N*‐acetylgalactosamine residues, linked to sialic acid residues, converting the diol units into aldehyde groups.^[^ ^136^, ^137^ ^]^ At the design stages of these approaches, one must also consider that the cell surface is constantly changing during the cell's lifetime, with glycocalyx elements being dynamically remodeled by hydrolases and glycosyltransferases.^[^ ^138^ ^]^ Exploiting these enzymes allows further manipulation of natural glyco‐elements through the insertion of unnatural saccharides or deletion of specific residues, redefining the naturally occurring carbohydrate repertoire. In this context, sialidase, a sialic acid hydrolase, that selectively cleaves sialic acid residues from cell surface glycans, has found numerous applications in cancer research, as these biomolecular effectors can counter the abnormal sialylation found in cancer cell surfaces, responsible for immune evasion.^[^ ^139^ ^]^ Importantly, during aldehyde generation, sialidase is generally used in combination with galactose oxidase, to cleave the glycoside linkage, thus providing a better exposure of the galactose/*N*‐acetylgalactosamine residues linked to the non‐reducing terminal of sialic acid.^[^ ^4^ ^]^ Such could be useful for further cell processing into programmable living assemblies that may take advantage of this added functionality to the cell surface.

Other relevant toolboxes for engineering cell assemblies are those comprising glycosyltransferases such as sialyltransferases and fucosyltransferases which have been widely used for glycan engineering due to the capability to insert a broad set of complex saccharides modified with non‐native moieties, directly in surface glycans, ultimately altering the cell's glycocalyx, and rendering it permissive to further processing.^[^ ^140^, ^141^, ^142^, ^143^ ^]^ Ligases, such as lipoic acid ligase, are another class of enzymes that find applications in native surface protein modifications. These enzymes naturally catalyze the addition of lipoic acid moieties to the lysine residues of specific proteins, finding interesting applications in the cell surface engineering field.^[^ ^131^ ^]^ By exploiting the plasticity of the binding site of such enzymes, cell surface proteins can be selectively modified by introducing both exogenous enzymes and functional substrate analogs, bearing new chemical handles (i.e., azides), that can be then incorporated and displayed by surface proteins.^[^ ^144^ ^]^

More recently, phenolic groups presented in naturally available tyrosine residues of surface proteins have been attracting attention as potential targets for enzymatic‐mediated modification, they can be readily conjugated with other phenolic moieties through the formation of di‐tyrosines, particularly through an exogenous enzymatic‐mediated oxidative process promoted by peroxidases.^[^ ^145^, ^146^ ^]^ The resulting covalent linkages are highly attractive for TERM applications and have already proven valuable for successfully installing biomaterials into mesenchymal stem cells (MSCs) surfaces, as it will be further presented.^[^ ^146^ ^]^

Enzymatic modifications through genetically inserted substrates are another valuable hybrid methodology that combines enzymatic‐mediated cell engineering with genetic engineering approaches. This method relies on a user‐programmable genetic insertion of a well‐defined recognition sequence into specific proteins, termed “fusion tag,” which, after expression will be used to identify the protein and serve as a substrate for enzymatic remodeling. The inserted fusion tag can be a protein‐ or a peptide‐tag, which will be recognized, allowing the direct attachment of modified materials or the incorporation of unnatural functional groups for further conjugation and engineering of cell‐rich assemblies.

In this toolset, halo‐tagging is an approach that relies on the expression of a protein‐tag, termed “Halo‐tag,” a mutant version of a bacterial haloalkane dehalogenase (Halotag protein (HTP)), that displays a mutation in its active site, enabling further cell surface engineering.^[^ ^133^ ^]^ This makes the enzyme unable to hydrolyze the intermediate carbon‐halogen bond formed between the enzyme and the halogenated substrates. By exploring this partially inactive enzyme, functional moieties bearing halogenated substrates can be trapped and irreversibly linked with the Halo‐tag, allowing a direct modification of the recombinant protein with great specificity and efficiency.^[^ ^147^, ^148^ ^]^ As this bacterial enzymatic reaction is foreign to mammalian cells, it is less likely to interfere and cross‐react with endogenous biochemical reactions.^[^ ^148^ ^]^ This type of protein‐tag is also known as a self‐labeling tag, as the protein/enzyme allows the direct attachment of a substrate to its structure, becoming a part of the whole modification inserted. Other self‐labeling protein‐tags, such as SNAP‐tag and CLIP‐tag have also been reported in cell surface engineering, especially for inducing artificial cell–cell contacts.^[^ ^128^, ^147^ ^]^ Yet, it is important to emphasize that protein‐tags are more likely to hamper protein function due to the protein's large size. Considering this, peptide‐tags are currently preferred, as they represent a minimal portion of all conjugate mass, reducing the final impact on recombinant protein's function. In this context, the use of transglutaminases, enzymes responsible for isopeptide bond formation between amino groups of lysine and terminal amine (‐NH_2_) groups of glutamine residues, is becoming highly attractive. By exploring this mechanism, surface proteins tagged with a 6 to 7 amino acid peptide‐tag (Q‐tag recognition sequence) can be modified with new moieties conjugated with amine groups, via transglutaminase enzyme activity.^[^ ^149^ ^]^ Peptide ligases are an attractive option for enzymatic remodeling as they allow the direct insertion of functional moieties bearing the enzymatic recognition sequence into the N‐ or C‐ terminal of the tagged protein, readily installing the desired modification onto the cell surface.^[^ ^3^ ^]^ For instance, the sortagging method that relies on a bacterial transpeptidase, Sortase A (SrtA), which recognizes a peptide‐tag and catalyzes the ligation of functional moieties bearing an LPXTG recognition motif and a recombinant protein expressing glycine repeats, has been recently explored for enzymatic‐mediated cell surface engineering.^[^ ^134^, ^150^ ^]^

On another perspective, biotin ligase can also be used as an alternative to biotinylate the cell surface through enzymatic approaches instead of relying on chemical or physical approaches. In this approach, a biotin ligase (BPL) is employed to catalyze the installation of biotin derivatives into cell surface proteins in the presence of ATP.^[^ ^135^, ^151^ ^]^ Different biotin ligases and corresponding mechanisms have been explored and are well described elsewhere.^[^ ^135^ ^]^ For instance, BirA, isolated from *E. coli* is the most well‐known example, promoting the biotinylation of the lysine residue within the peptide‐tag comprised by a 15 amino acid motif (Biotin Acceptor Peptide tag (BAP) tag), expressed in the protein of interest.^[^ ^39^ ^]^ Protein‐tags can also be used for enzymatic biotinylation, such as biotin carboxyl carrier protein, which is recognized by a biotin ligase from *Sulfolobus tokodaii*.^[^ ^152^ ^]^

The great plethora of different enzymes and mechanisms expands the possibilities for modifying the cell surface. Several other studies are contributing toward increasing our body of knowledge on this technique and opening new avenues for exploring it for engineering living assemblies.^[^ ^134^, ^135^, ^147^, ^153^ ^]^

#### 2.2.3 Metabolic Engineering

Metabolic engineering methods exploit the intrinsically active cell metabolism and native biosynthetic machinery to install a relatively small chemically functionalized precursor in different biomacromolecules on the cell surface. One of the most widely explored metabolic‐based approaches encompasses the exploitation of the glycan biosynthetic machinery, a method termed—metabolic glycoengineering (MGE). This highly biocompatible approach allows a transient remodeling of cells’ glycocalyx with natural or unnatural functional groups, upon incorporation of modified monosaccharides into specific metabolic pathways.^[^ ^154^ ^]^ While the enzymatic remodeling of glycans enables the direct introduction of complex saccharides, metabolic glycoengineering relies on small and simple monosaccharide analogs that are processed in multiple enzymatic steps as these are recognized as naturally occurring species. This approach critically depends on the enzymes of the explored metabolic pathway and is generally used to install relatively small chemical moieties on the cell surface.^[^ ^155^, ^156^ ^]^ Within the great variety of monosaccharides found in glycoconjugates, terminal monosaccharides, including sialic acid (Sia), d‐galactose (Gal), l‐fucose (Fuc) residues, all represent an interesting modification point for precision engineering of the cell surface glycocalyx.^[^ ^155^ ^]^ Currently, *N*‐acetylneuraminic acid (Neu5Ac), a known member of the sialic acid family, has been the foremost target in the majority of MGE applications. Consequently, the sialic acid biosynthetic pathway, including the Roseman‐Warren pathway, which describes the de novo biosynthesis of this residue, has also been the subject of several studies.^[^ ^155^ ^]^ With recent advances, different monosaccharide analogs were introduced in cell surface glycocalyx, providing new aliphatic or bioorthogonal modifications.^[^ ^40^, ^155^, ^156^, ^157^ ^]^ Aliphatic analogs exhibit chemically inert modifications, where the *N*‐acyl is simply elongated with methylene groups.^[^ ^155^ ^]^ This *N*‐acyl elongation ultimately results in intriguing alterations in biological processes such as cell adhesion, and neuronal differentiation, among others.^[^ ^155^, ^158^, ^159^, ^160^ ^]^ However, when considering cell surface engineering applications, bioorthogonal analogs have been the most attractive in recent applications of this methodology for engineering cell–cell and cell–material assemblies.^[^ ^21^ ^]^ Particularly, after the introduction of a ketone group by the incorporation of the first successful monosaccharide analog, *N*‐levulinoylmannosamine (ManNLev), distinct functional groups have been installed, creating a vast plethora of functional libraries of monosaccharides analogs that researchers can select to precisely install natural or unnatural chemical functionalities in the cell glycocalyx.^[^ ^9^, ^161^, ^162^, ^163^, ^164^, ^165^ ^]^ These displayed functional moieties can then be combined with a great variety of larger and more complex moieties, reinforcing the versatility of this technique.^[^ ^166^ ^]^ MGE is generally performed under mild conditions and applies virtually to any type of mammalian cell. However, the functionalization efficiency can vary depending on the delivered dose, cell type, and the size/chemical type of the inserted modification.^[^ ^167^ ^]^ Another important advantage is the relatively short lifetime of the inserted modification. This characteristic is highly related to the faster rate of turnover of peripheral sugar residues, including Neu5Ac, when compared with other core sugars, which allows the mitigation of potential long‐term effects in cell function/behavior caused by the structural modification of sugar residues.^[^ ^155^ ^]^

Metabolic engineering through unnatural monosaccharides as metabolic precursors has been the most used metabolic strategy for cell surface engineering. Additionally, metabolic engineering of lipids through modified lipid analogs feeding constitutes an interesting and promising strategy for surface engineering. These biologically relevant molecules represent a considerable portion of the cell surface, and thus, constitute an attractive spot to introduce chemical groups that can be further conjugated with additional functional moieties. Functional moieties can be inserted either into the fatty acyl tails or headgroups of the lipid. The terminus of the acyl chain is often used to introduce functional moieties upon fatty acid analogs feeding. However, numerous types of lipids can incorporate these fatty acids into their structure, which reduces labeling specificity.^[^ ^131^, ^168^ ^]^ Considering this, lipid headgroups are a more interesting and promising target for metabolic labeling, once they are installed through more exclusive biosynthetic pathways, providing a certain degree of specificity, and are presented to the external environment in the outer leaflet. Still, the ability to explore metabolic lipid engineering in a headgroup‐specific manner is hindered by the complexity of the lipid metabolic pathway, resulting in a small number of reported successful modifications.^[^ ^169^ ^]^ Typically, choline analogs are used for the incorporation of functional handles into choline‐containing lipids, such as phosphatidylcholine and sphingomyelin, by leveraging phospholipase D (PLD) bioactivity.^[^ ^170^, ^171^ ^]^ PLD is naturally responsible for the hydrolysis of phosphatidylcholine into phosphatidic acid. However, in the presence of exogenous primary alcohols that bear the desired chemical moiety, this enzyme catalyzes a transphosphatidylation, resulting in a functionalized phosphatidyl alcohol. Examples of common choline analogs include propargylcholine, azidoethylcholine, alkynols, and, azidoalcohols.^[^ ^170^, ^171^, ^172^, ^173^ ^]^

Gathering on the plethora of available cell surface engineering technologies, the selection of a specific methodology for generating cell‐rich materials should be carefully addressed taking into consideration several parameters including the types of biomolecular targets, the surface functionalization lifetime, and the cytocompatibility of the methodology to be employed (**Table** **1**). Ultimately, all of these can impact the production of living cell assemblies and their biofunctionality. The exploitation of advanced characterization techniques (i.e., omics‐based approaches),^[^ ^174^, ^175^ ^]^ may provide a deeper insight into short‐ and long‐term effects in modified cells, further aiding researchers in the selection of a specific technology at early design stages.

**Table 1:** Summary of engineering technologies for cell surface modification.

CSE Toolsets		General approaches	Advantages	Limitations	Targets	Cytocompatibility	Functionalization lifetime	Ref.
Chemically‐driven	Covalent‐based	Direct (amine‐ and thiol‐based) Indirect (aldehyde‐based)	Reactive groups availability Simple modification	Potential physiological alterations and loss of surface protein activity Required chemical preconditioning (indirect approaches) Lack of target selectivity	Proteins Glycans	Medium/high Chemistry dependent (e.g., maleimide, NHS, hydrazine‐based reactions)	Up to 4 days (thiol‐based approach)^[^ ^49^ ^]^	[15, 53, 78]
	Electrostatic‐mediated	Cationic polymers and nanoparticles	Simple and cost‐effective	Cell damage upon internalization Extensive coating may impact cell bioactivity Time‐consuming process (e.g., layer‐by‐layer approaches) Lack of target selectivity	Any charge complementary cell surface biomolecules	Medium Biomolecule and nanoparticle dependent (e.g., polymer type MW, polymer, or nanoparticle charge, etc.)	<30 min (synthetic polycation—PLL)^[^ ^80^ ^]^ Relatively prolonged exposure (>7 days, natural biopolymer)^[^ ^61^ ^]^	[58]
	Hydrophobic insertion	Lipidic molecules (synthetic phospholipids, cholesterol anchors, etc.) Fusogenic liposomes	Ease and rapid anchoring Minimal impact on cell's viability and functions Lipids' chemical versatility	Rapid and passive dissociation Possible internalization Lack of target selectivity	Phospholipid bilayer	Medium/high Lipid dependent (e.g., tendency for internalization, fluidization of cell membrane, etc.)	>1.5 h (multivalent cholesterol anchors)^[^ ^76^, ^77^ ^]^ Up to 7 days (liposome fusion)^[^ ^10^ ^]^	[53, 73]
	Biomolecular recognition	Antibodies Aptamers Targeting peptides	Target selectivity and programmability High binding affinity	Chemically complex modifications Potential immunogenic responses (antibodies)	Proteins Glycans Lipids	Medium/high Dose‐dependent	Minutes to hours (depending on specific affinity) *t*_1/2_ = 10 min^[^ ^176^ ^]^	[84, 88]
Biologically‐driven	Genetic engineering	Nucleic acids’ delivery (i.e., insert genetic circuits, optogenetic tools, etc.)	Biological programmability Spatiotemporal control Target selectivity	Risk of insertional mutagenesis Risk of trigger immunogenic responses and tumorigenesis Cell‐type dependency	Proteins	Medium/high Long‐term effects to be fully determined Delivery method dependent	Cell lifetime (stable expression)^[^ ^120^ ^]^	[5, 109, 121, 126]
	Enzyme‐mediated	Natural substrate conversion (galactose oxidase, sialidase, lipoic acid ligase, etc.) Genetically‐inserted substrate conversion (Halo‐tag, SNAP‐tag, transglutaminase, sortase, etc.)	Site‐specificity High target selectivity	The majority of the methods rely on fusion tags High‐cost approach Relatively low enzyme stability	Proteins Glycans	Medium/high Chemical reaction‐ and enzyme‐dependent	Up to 8 days^[^ ^177^ ^]^	[3, 5, 135]
	Metabolic engineering	Glycoengineering Lipid engineering	Diversity of chemical groups that can be introduced Modification transience (short functionalization lifetime) High target selectivity	Limited to insertion of relatively small chemical moieties Chemical insertion efficiency can vary depending on cell metabolism	Glycans Lipids	Medium/high Chemical moiety‐ and dose‐dependent (long‐term effects to be fully determined)	Up to 7 days^[^ ^178^ ^]^	[71, 155, 179]

The use of such cell surface engineering techniques for fabricating cell–cell and biomaterial‐driven, quasi all‐cellular living assemblies will be showcased and critically discussed in the following sections, from the perspective of their potential biomedical applications.

## 3 Programming Cellular Interactions for Engineering Living Assemblies

**Figure 2:**
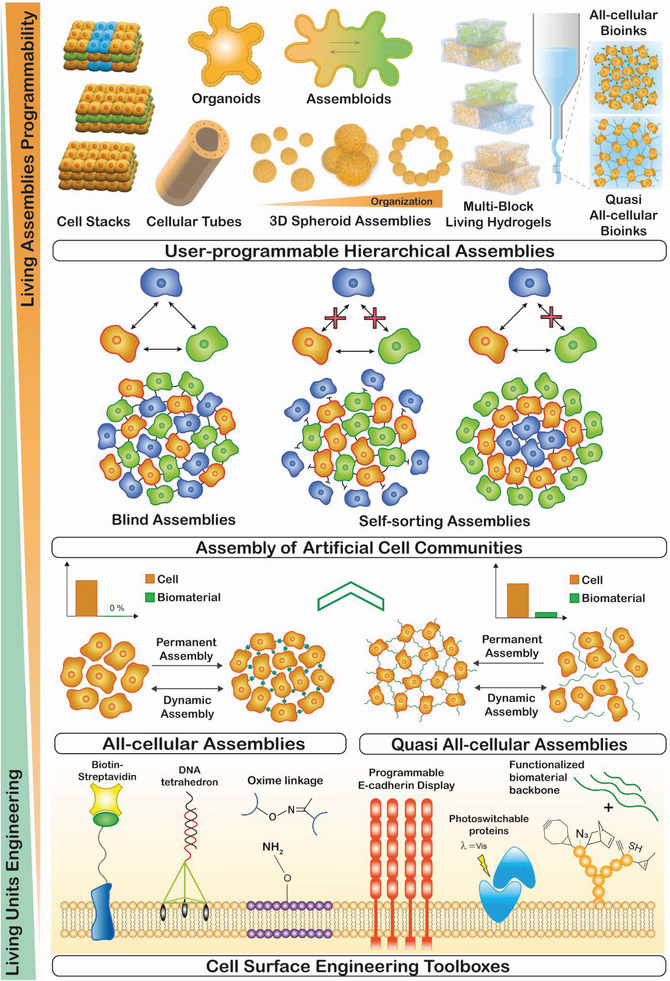
Schematic representation of bottom‐up engineered living materials with increasing hierarchy and biological functionalities that are enabled by engineering the surface of cellular building blocks. In the design stages of surface‐engineered cells, researchers can leverage chemically‐ or biologically‐driven toolboxes for generating functional cell units with added processability and biofunctionality. The added functionalities on the cell surface potentiate their programming into permanent or dynamic living cell assemblies where cell–cell interactions are mediated by surface functionalization. In addition, cell surface functionalization can also enable their conjugation to cell‐tethering biomaterials (i.e., polymers, proteinaceous materials, etc.) creating quasi all‐cellular monotypic or heterotypic assemblies. Functionalization with specific groups enables researchers to program increasingly complex living cell communities comprising heterotypic cellular elements with self‐sorting and self‐organizing capabilities. These can in turn be processed into living assemblies at different length scales and with different architectural and dynamic biological features.

During organogenesis and morphogenesis, a strong interplay between cells and the extracellular elements takes place to elegantly self‐orchestrate multicellular assemblies, presenting robust and dynamic cell–cell and cell–matrix interactions. In these living assemblies, the established biochemical and biophysical interactions are tightly regulated and remodeled in response to intracellular and extracellular cues.^[^ ^26^ ^]^ Aiming to recapitulate such natural building blocks and their dynamic interplay, bottom‐up bioengineering approaches have already provided robust platforms to develop complex 3D cell‐rich architectures with well‐defined biological functions and spatiotemporal evolvability, closely recapitulating key aspects of native multicellular assemblies in tissues and organs (**Figure** **2**).^[^ ^21^, ^26^ ^]^

Cell surface engineering toolboxes introduce new possibilities to promote the programmable self‐assembly of functional unitary building blocks into higher‐order complex architectures, from the bottom‐up. As above discussed, so far, different functional moieties have been introduced in the cell surface to directly promote interactions or to act as an anchoring point for conjugating intermediary elements capable of recognizing and connecting multiple cells, thus enabling a precise control over cell–cell and cell–biomaterial interactions.^[^ ^69^ ^]^ Through rationally designing the cell surface, such functionalized cellular building blocks can be spatiotemporally molded and processed for the establishment of robust and complex 3D bioarchitectures with living features. The following section provides an outlook of state‐of‐the‐art cell surface engineering approaches used for programming cell–cell and cell–biomaterial assemblies, highlighting their potential as bottom‐up tissue engineering platforms to address complex biologic problems.

### 3.1 Programmable Cell–Cell Assemblies

Cell–cell interactions are critical during the development of multicellular organisms, driving the well‐orchestrated self‐organization of complex architectures. In nature, this type of intercellular communication is strongly dependent on cell–cell aggregation events, which are achieved through the interactions of particular receptors displayed on the cell surface. These tightly modulate cell recognition and communication in the intercellular environment, ultimately guiding a proper spatial and temporal organization of multicellular assemblies.^[^ ^34^, ^69^ ^]^ As above evidenced, up‐to‐date, researchers have devised powerful alternatives to mimic these receptor/ligand mechanisms and artificially manipulate cell–cell aggregation to establish self‐organized assemblies with dynamic properties and biomimetic biofunctionalities, expanding the bottom‐up tissue engineering field. Aiming to precisely control such interactions, researchers explored different cell surface engineering methods that have been applied to redecorate cell surfaces with potential artificial receptors. As discussed in the following examples, cell–cell assemblies could be driven directly by the conjugation between chemical groups naturally or unnaturally displayed on the cell surface, or, as shown in recent cases, through an intermediary of high‐order functional elements (i.e., DNA, proteins/peptides, host‐guest pairs) anchored onto the cell surface, achieving an enhanced control and selectivity over the onset of the interactions.

**Figure 3:**
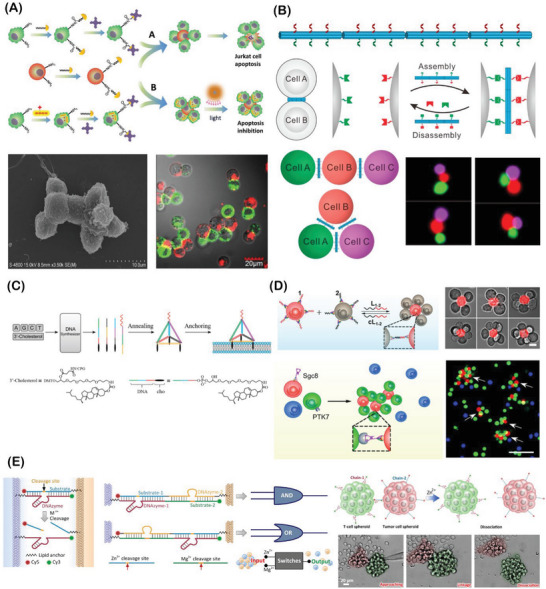
Covalent and hydrophobic modification of the cell surface. A) Schematics of cell surface amine groups functionalization with the biotin‐streptavidin system to induce a multicellular assembly of T cell leukemia cells (Jurkat) and natural killer cells (NK‐92MI). SEM (lower left) and confocal laser scanning microscopy (CLSM, lower right) micrographs of multicellular assemblies, cell ratio: 1:1. Scale bars: 20 µm and 10 µm, respectively. Reproduced with permission.^[^ ^180^ ^]^ Copyright 2014, Wiley. B) Schematics of organized cell–cell assemblies through the intermediary of DNA origami nanostructures attached to cell surface amines. Fluorescence images (lower right) of Jurkat origami clusters with linear (left) and closed‐ring (right) topology. Reproduced with permission.^[^ ^181^ ^]^ Copyright 2020, American Chemical Society. C) Illustration of DNA tetrahedron fabrication and cell surface functionalization by hydrophobic insertion of cholesterol anchors. Reproduced with permission.^[^ ^76^ ^]^ Copyright 2021, American Chemical Society. D) Schematics of DNA tetrahedron‐based cell–cell assemblies with controlled and selective properties. CLSM micrograph of intercellular interactions promoted by DNA hybridization (upper) in CCRF‐CEM cells population and aptamer‐based targeting (lower) in heterotypic populations of K562, CEM, and Ramos cells; cell ratio: 1:10:10. Scale bars: 10 µm and 100 µm, respectively. Reproduced with permission.^[^ ^77^ ^]^ Copyright 2019, American Chemical Society. E) Schematic illustration of hydrophobic installation of DNAzymes to manipulate cell–cell interactions via a two‐factor‐based system. Bright‐field images of T‐cell spheroid reveal a Zn^2+^‐dependent assembly/disassembly process toward tumor cell spheroid. Scale bar: 20 µm. Reproduced with permission.^[^ ^182^ ^]^ Copyright 2021, American Chemical Society.

Direct chemical covalent modification of naturally available amine groups has been one of the most straightforward approaches to installing new functional moieties onto cell surfaces. Leveraging this cell surface engineering tool, researchers developed a platform to screen the interplay between immune and cancer cell assemblies.^[^ ^180^ ^]^ For this, cell surface biotinylation was performed through the covalent attachment of a biotinylated sulfo‐*N*‐hydroxyl‐succinimide into the available surface amines, successfully installing biotin molecules onto the cell surface.^[^ ^180^ ^]^ Due to the strong biotin‐streptavidin molecular affinity, biotin molecules displayed on the cell surface were conjugated with streptavidin molecules, allowing the establishment of stable cell–cell linkages and the formation of heterotypic multicellular assemblies comprised by immune cells, namely NK‐92MI and Jurkat T‐cells (**Figure** **3**A). The promoted spatial proximity between cells resulted in improved antitumoral activity of immune cells toward cancer cells, revealing a spatial regulation of cell–cell communications within the multicellular assembly. Moreover, researchers were able to obtain temporal control over cell–cell communication through the light‐induced formation of reactive oxygen species, controlling the apoptotic activity within the multicellular assembly (Figure 3A). Besides surface biotinylation, DNA origamis resembling a common DNA nanostructure have also been used for cell surface decoration.^[^ ^84^ ^]^ Recently, the potential of DNA origami nanostructures (DON) for mediating cell–cell interactions was explored through the covalent modification of amine groups to display thiol groups for further conjugation with thiolated ssDNAs.^[^ ^181^ ^]^ Cell–cell interactions were then established through intermediary DNA origami structures modified with numerous complementary ssDNA strands, allowing the formation of organized homotypic and heterotypic cellular assemblies through selective DNA hybridization (Figure 3B). This approach showed the potential of exploiting DON for the development of cell clusters with programmable spatial/geometric configurations, as well as the possibility of developing asymmetric arrangements. Such technology can be particularly interesting in promoting contact‐induced cell–cell communications that can be useful for screening advanced cellular therapies, including immunotherapies.

Besides the attachment of DNA origami, DNA tetrahedrons were also successfully installed into the cell surface through lipid anchoring, showing tunable affinity, as well as great anchor stability even in the presence of serum proteins, revealing slower endocytic kinetics when compared with simple amphiphilic ssDNA (Figure 3C).^[^ ^77^ ^]^ It is important to emphasize that hydrophobic insertion through lipid anchoring is the most commonly used technique to install DNA nanostructures on the cell surface.^[^ ^84^ ^]^ In this context, self‐assembled DNA tetrahedrons, formulated by four special DNA oligonucleotides, were anchored into the cell surface through cholesterol molecules, culminating in an amphiphilic pyramidal DNA structure that showed outstanding stability and anchoring efficiency. Such stability resulted in a delay in the structure's dissociation from the cell membrane. Using this method, cell–cell attachment within two cell batches bearing different DNA sequences could be precisely controlled via DNA hybridization upon the introduction of a DNA linker as an intermediate. Upon introduction of a fully complementary DNA of the inserted linker into the system, cell assembly can be reverted (Figure 3C). In the same study, cell–cell adhesion was also promoted through the introduction of a specific aptamer into the tetrahedron apex. Using this approach, K562 cells engineered with the aptamer sgc8 were able to attach specifically to target CEM cells, leaving non‐targeted cells aside, resulting in a cell‐type specific intercellular assembly (Figure 3C).^[^ ^77^ ^]^ Considering this, such dynamic interactions may be interesting to explore in different therapies, especially in the study and development of immunotherapies. In an attempt to obtain a stimulus‐driven cell–cell interaction, an anti‐ATP aptamer was introduced into the apex of the DNA tetrahedron, blocking the DNA hybridization, so that the cell–cell interaction only occurs when ATP is detected by the blocking aptamer, mimicking the allosteric modulation found in biological systems (Figure 3D).^[^ ^76^ ^]^ Although the resulting platform was not directly applied for TERM applications, this blocker could be beneficial for the improvement of the temporal resolution and dynamic of the structures assembled by using amphiphilic DNA tetrahedrons. With the recent improvements made in the DNA technology area, new functional nucleic acids (FNA), including DNAzymes, were anchored to the cell surface through hydrophobic insertion using cholesterol anchors.^[^ ^182^ ^]^ These special DNA sequences exhibit a metal ion‐dependent catalytic activity, being able to selectively cleave specific DNA/RNA sequences, enabling control over intercellular interactions upon metal ion trigger.^[^ ^183^ ^]^ With this in mind, researchers were able to establish reversible cell–cell assemblies via hybridization. These could be controlled through metal ions in a two‐factor‐based system, allowing an effective manipulation of cell–cell interlinkages and their dynamic behaviors (Figure 3E).^[^ ^182^ ^]^ In addition, this concept was successfully leveraged for spheroids aggregation, which also resulted in improved T‐cell spheroid migration toward different tumor cell spheroids, revealing great versatility to control cell–cell interactions and establish dynamic cell systems for immunotherapies.

**Figure 4:**
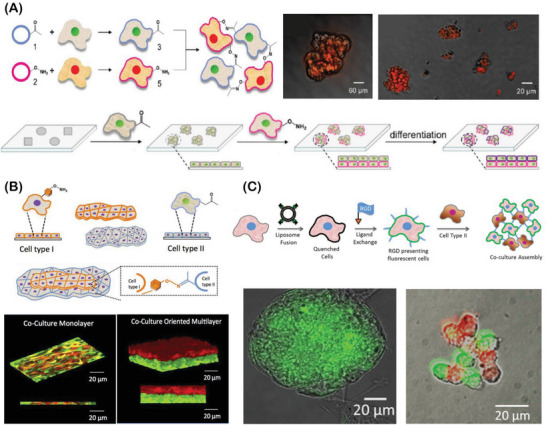
Hydrophobic functionalization of cell surface via liposome fusion. A) Schematic illustration of cell–cell aggregation and multilayered microtissue formation via bioorthogonal oxime linkage between ketone and oxyamine groups installed into the cell surface by fusogenic liposomes. Overlay fluorescent and phase contrast images of spheroids by crosslinking between oxyamine and ketone groups tethered on RAT‐2 cell line and 3T3 fibroblasts. Scale bars: 60 µm (left) and 20 µm (right). Reproduced with permission.^[^ ^10^ ^]^ Copyright 2011, American Chemical Society. B) Multilayered 3D structures assembled via oxime linkage between photo‐oxyamine functionalized hMSCs and ketone‐functionalized fibroblasts. Reproduced with permission.^[^ ^33^ ^]^ Copyright 2014, Springer Nature. C) Schematics of a dual‐step methodology for controlled cell assembly, via in situ ligand exchange and RGD‐integrin recognition. Overlay of fluorescence and phase contrast images of bioengineered fibroblast clusters. Scale bar: 20 µm. Reproduced with permission.^[^ ^187^ ^]^ Copyright 2015, American Chemical Society.

Alongside the formation of spherical or random aggregates, researchers have found ways to pattern cells using DNA‐based platforms that allow the programming and tuning of different microtissue features such as size, shape, composition, and spatial heterogeneity. This enables the possibility of studying the effect on cell behavior within multicellular architectures, revealing great potential to be used for recapitulating the complex cellular arrangements observed in native tissues.^[^ ^184^ ^]^ Heterotypic cell populations were decorated using lipid‐DNA conjugates and selectively conjugated via DNA hybridization between complementary strands. DNA‐patterned surfaces were used as the initial template for specific and localized cell attachment. This was followed by layer‐by‐layer assembly of cells bearing complementary sequences, enabling spatial organization with single‐cell resolution. The outstanding spatial resolution allowed for the fabrication of organoid‐like microtissues with tunable spatial heterogeneity and organization. Besides being a valuable platform to better understand the correlation between tissue morphology and cell behavior, such a platform may also be explored for tissue repair and/or disease modeling applications. Interestingly, within hydrophobic insertion platforms, functionalized fusogenic liposomes can be efficiently incorporated into the target cell, thus displaying their phospholipidic content on the cell surface. After a successful installation of ketone groups into the cell surface through liposome fusion, Yousaf's group explored this technology for generating 3D tissue‐like assemblies.^[^ ^185^ ^]^ Initially, researchers reprogrammed two populations of fibroblasts with ketone and oxyamine groups, that were able to undergo intercellular crosslinking via bioorthogonal oxime‐based click‐chemistry, rapidly generating 3D spheroid assemblies, as well as larger 3D multilayered tissue‐like assemblies (**Figure** **4**A).^[^ ^10^ ^]^ More recently, liposome fusion technology was applied for establishing organ‐like assemblies by co‐culture of three reprogrammed cell lines engineered using this strategy.^[^ ^32^, ^186^ ^]^ Particularly, physiomimetic 3D liver tissue models were established upon an agglomeration of distinct cell lines, including hepatocytes, hepatic endothelial cells, and hepatic stellate cells, resulting in a versatile in vitro model to be explored for drug discovery and screening.^[^ ^186^ ^]^ Similarly, a scaffold‐free 3D cardiac tissue model was also assembled by combining three distinct cell lines through oxime click reaction.^[^ ^32^ ^]^ The tissue‐like structure successfully exhibited proper biological functions, revealing a spontaneous and synchronous beating along the fabricated tissue. This approach reveals great promise to be further improved by using biofabrication tools to fabricate larger and clinically‐relevant engineered tissues for TERM or in vitro drug screening applications. In an attempt to improve the spatiotemporal resolution of their structures, researchers explored a different methodology and installed a photo‐cleavable oxyamine moiety able to undergo cleavage upon mild ultra‐violet (UV) irradiation, prompting the disassembly of the multilayered 3D cell structures (Figure 4B).^[^ ^33^ ^]^ By exploring an in situ ligand exchange reaction, the cell surface was modified in a double‐step method resorting to liposomes containing a quenched calcein dye linked to dabcyl hydrazine (quencher), that may be exchanged by an oxyamine‐containing RGD peptide through RGD‐integrin recognition, providing new approaches for controlling tissue assembly and organization owing to RGD‐integrin interactions (Figure 4C).^[^ ^187^ ^]^

Apart from these approaches, the cell surface is also well decorated with naturally displayed chemical groups that can be exploited as targets for directing surface functionalization with acceptable specificity. Molecular recognition elements are commonly employed as intermediary agents to engineer the cell surface by promoting cell targeting through the recognition of specific ligands, allowing the recapitulation of self‐sorting events occurring within multicellular architectures. Recent approaches are implementing the concept of molecular recognition to directly promote cell–cell aggregation through aptamer‐target and antigen‐antibody interactions. Relying on the highly selective aptamer‐target interaction, bispecific multivalent aptamer structures were synthesized and installed into the cell surface through specific recognition of ligands displayed on Ramos (RA 1) and Jurkat cell's surface. A five‐point‐star DNA nanostructure scaffold was essential to assemble and coordinate the arrangement of aptamers within the structure, allowing for a controlled design over the valence, orientation, and distance of the displayed aptamers, resulting in an increased binding affinity. The ensuing structure not only allowed the simultaneous decoration and linkage of two cell types but also showed an improved binding affinity, superior to monovalent and linear platforms.^[^ ^188^ ^]^

**Figure 5:**
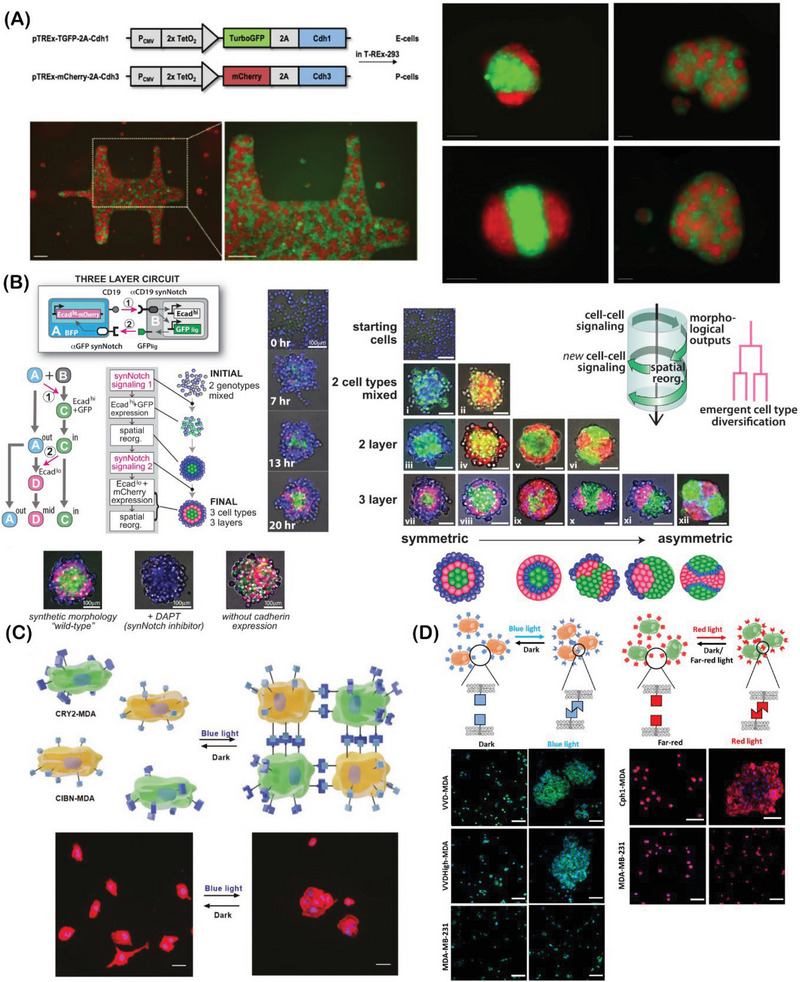
Cell surface manipulation via genetic engineering tools. A) Fluorescence micrograph of cadherin‐based 2D (left) and 3D (right) patterning system. Imaging from 3D T‐Rex‐293 cell patterns generated in aggregates of 1 000 (left column) and 10 000 (right column) cells. Scale bars: 200 µm. Reproduced under the terms of CC‐BY Creative Commons Attribution 4.0 International Licensehttp.^[^ ^189^ ^]^ Copyright 2016, The Authors, Published by Springer Nature. B) Schematics of self‐organizing spheroids established by genetically incorporated three‐layer circuits in mouse L929 fibroblasts. Structures exhibit intricate cell–cell signaling arising from the synNotch‐adhesion system, leading to a self‐modulation of the spatial organization and consecutive structural symmetry breaking. Reproduced with permission.^[^ ^34^ ^]^ Copyright 2018, American Association for the Advancement of Science (AAAS). C) Schematics of a blue‐light switchable cell–cell assembly based on the heterodimerization of CRY2 and CIBN. Fluorescence micrographs from the light‐responsive clustering process based on CRY2‐CIBN heterodimerization. Scale bar 50 µm. Reproduced with permission.^[^ ^119^ ^]^ Copyright 2019, Wiley‐VCH. D) Orthogonal cell–cell assembly based on VVD and Cph1 expression in MDA‐MB‐231 breast cancer cell populations. Homodimerization occurs under blue‐ and red‐light irradiation, respectively, during 4h. Scale bars: 200 µm. Reproduced with permission.^[^ ^120^ ^]^ Copyright 2020, American Chemical Society.

In the context of controlling the cell surface to promote specific cell–cell interactions, synthetic biology is a particularly valuable toolbox that enables researchers to completely alter the cell surface by introducing new proteins, lipids, and carbohydrates, or by modifying the expression/activity of existing molecules. For instance, synthetic biology has been used to engineer cells with novel adhesion properties that increase/decrease adhesion to specific cell types. These strategies can also be leveraged to direct cell behavior in a user‐programmable manner, such as directing cells to establish specific interactions or patterned features, which can be directly explored as building blocks to engineer living tissues with complex and tunable cellular organizations. Following this rationale, synthetic biology tools have supported the recent development of a phase separation system mediated by differential cadherin expression for application in larger multicellular populations.^[^ ^189^ ^]^ In the developed system, two morphogenetic modules responsible for the expression of P‐cadherin or E‐cadherin were separately incorporated into individual T‐Rex‐293 cell populations. Due to a stronger cell–cell adhesion driven by homotypic interactions, cellular patterning events were verified, allowing self‐sorting to take place during cellular assembly. This demonstrates the potential of the cadherin‐mediated self‐sorting system to generate complex patterns de novo, mimicking key aspects of developmental biology during the bottom‐up assembly of complex living architectures (**Figure** **5**A). Following a similar concept, researchers have recently developed new ways to manipulate cell–cell interactions by genetically installing synthetic cell adhesion molecules (synCAMs).^[^ ^190^ ^]^ These newly engineered cell adhesion molecules (CAM) were obtained by replacing the native ECD with an orthogonal ECD, providing greater control over the selectivity and affinity of cellular interactions. The resulting set of combinations between orthogonal ECDs and endogenous CAM intracellular domains provides a versatile toolbox for programming cell–cell interfaces. This programming includes the possibility of building modular multicellular assemblies that can then be leveraged to form large‐scale tissues with complex features. Moreover, researchers observed integrative properties, revealing that synCAM‐engineered cells were capable of intercalation and remodeling of previously established architectures formed by native CAMs. Relying also on cadherin‐mediated cell–cell adhesion, synNotch genetic circuits were genetically installed in cells to modulate cell–cell signaling networks and promote self‐organization within cell populations.^[^ ^34^ ^]^ Starting with a simpler two‐cell‐type test, a synNotch receptor capable of recognizing CD19 ligands from a sender cell population and consequently triggering the downstream activation of E‐cadherin expression, was genetically installed onto the surface of the receiver cell population. This resulted in a programmed two‐layered living architecture owing to the orchestrated cell sorting process (Figure 5B). Upon the addition of a synNotch signaling blocker (DAPT), E‐cadherin induction was inhibited, preventing cell sorting and impeding multicellular structures’ assembly. Moreover, more complex cellular assemblies were set up by introducing different synNotch programs to the equation. The new synNotch receptor displayed in sender cells detects surface‐tethered GFP expressed by previously activated receiver cells, resulting in the expression of low quantities of E‐cadherin. The differential expression of E‐cadherin promoted the self‐assembly of a three‐layered cellular construct comprised of three distinct cell phenotypes (Figure 5B).^[^ ^34^ ^]^ Even more, by changing the design of the system, an asymmetric morphologic spatial reorganization based on cadherin affinity was obtained, yielding a living architecture that mimics the symmetry breaking found in natural morphogenesis events (Figure 5B). Optogenetic tools have undoubtedly provided the community with a higher spatial and temporal control over the cellular assemblies, opening new ways to manipulate their assembly process. In an elegant approach, the genetic installation of blue‐light switchable proteins, CRY2 and CIBN, was used as artificial adhesion molecules installed into the surface of distinct MDA‐MB‐231 breast cancer cell populations, allowing to attain photo‐control over cell–cell interactions.^[^ ^119^ ^]^ Relying on the heterodimerization between these two complementary proteins, researchers were able to promote a dynamic assembly of cells through the formation of heterophilic cell–cell interactions, upon blue‐light irradiation. These interactions could be easily disassembled in the darkness, capturing the natural reversibility and dynamic nature of cell–cell interactions (Figure 5C). In a final test, researchers were able to explore the potential of photo‐switchable cells as the building blocks of a layered 3D architecture. These exclusively form under blue‐light irradiation, highlighting the versatility of these technologies to be leveraged for the fabrication of multicellular architectures with tunable self‐organizational levels. In a follow‐up study, three different photo‐switchable protein pairs exhibiting distinct protein‐protein interaction dynamics, kinetics, and strengths were explored to modulate cell self‐assembly dynamics and self‐sorting events.^[^ ^126^ ^]^ To accomplish this, distinct MDA‐MB‐231 breast cancer cell populations were engineered with protein pairs, iLID/Nano, nMag/pMag, nMagHigh/pMagHigh, that heterodimerize under blue‐light irradiation, promoting cell aggregation, and that revert in the absence of light. By applying different light pulse sequences, different protein‐protein interactions can be explored for modulating social‐like cell–cell assembly dynamics. The resulting platform proved the possibility of applying concepts from colloidal systems to the bottom‐up fabrication of artificial cell communities while providing a broad set of tunable parameters (i.e., light wavelength, protein‐protein pairs, etc.). More recently, the potential of optogenetics for differential self‐assembly within multitype cell populations has been explored.^[^ ^120^ ^]^ For this, distinct photo‐switchable proteins, VVD and Cph1, were genetically installed into the surface of MDA‐MB‐231 cells, resulting in two different surface‐engineered cell populations. In the presence of blue‐light (for VVD) or red‐light (for Cph1), the encoded photo‐switchable proteins homodimerize, allowing for the establishment of selective and homophilic reversible cell–cell interactions (Figure 5D). In addition to achieving a dynamic and reversible self‐assembly mechanism, the resulting cellular assembly was able to replicate the biological self‐sorting events of different cell types, similar to what is observed in organogenesis and morphogenesis processes. Regarding these capabilities, such tools may be advantageous for developing advanced building blocks that can be processed by biofabrication into complex living materials that are capable of self‐organizing according to a developmental biology rationale in order to recapitulate the complex architectural and cellular arrangements of native living tissues.

**Figure 6:**
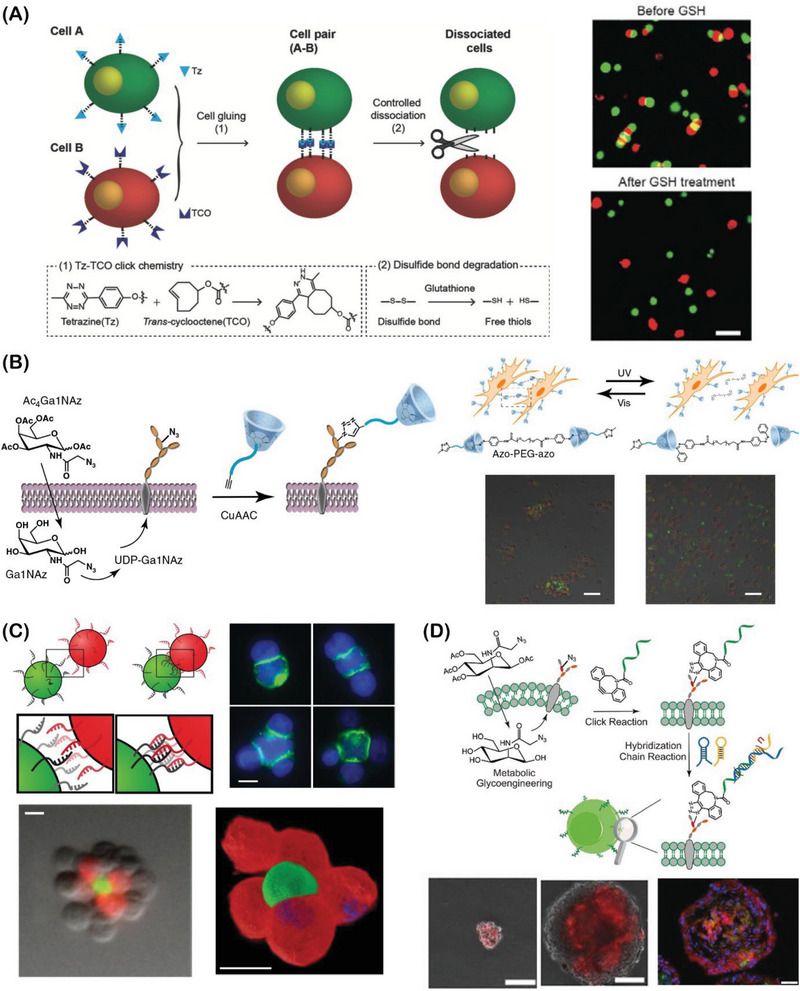
Cell–cell assemblies established via metabolically‐engineered cell surfaces. A) Schematic of reversible cell aggregation systems, based on a double‐step strategy combining metabolic glycoengineering and double click‐chemistry approaches. Fluorescence micrographs of engineered Jurkat T‐cells show disulfide bonds’ degradation in the presence of glutathione, resulting in the quick detachment of cell aggregates. Scale bar: 30 µm. Reproduced with permission.^[^ ^192^ ^]^ Copyright 2016, American Chemical Society. B) Schematic illustration of metabolic incorporation of azide groups for cell surface functionalization with an intermediary host‐guest system to attain a light‐responsive assembly/disassembly process. CLSM micrographs displaying light‐responsive intracellular interactions between red‐ and green‐stained β‐CD‐modified MCF‐7 cells (cell ratio 1:1). Scale bars: 50 µm. Reproduced under the terms of the CC‐BY Creative Commons Attribution 4.0 International License (http://creativecommons.org/licenses/by/4.0/).^[^ ^104^ ^]^ Copyright 2016, The Authors, Published by Springer Nature. C) Schematic of controlled cell–cell assemblies via hybridization of ssDNA chemically attached to metabolically labeled Jurkat T‐cells. Fluorescence micrographs showing evidence of cell–cell assembly, revealing ssDNA hybridization at the intercellular interface. Scale bars: 10 µm. Reproduced with permission.^[^ ^194^ ^]^ Copyright 2009, National Academy of Sciences. D) Schematic of in situ formation of polyvalent DNA nanostructures based on the initial chemical conjugation of DNA with metabolically installed azide handles. Fluorescence images of the size analysis of heterotypic multicellular assemblies comprised of hMSC and NHAC cells, between day 1 (left) and day 20 (middle). Immunofluorescence micrograph revealing type II collagen and aggrecan presence. Scale bars: 100 and 50 µm, respectively. Reproduced with permission.^[^ ^195^ ^]^ Copyright 2018, Wiley.

Adding to the possibility of using genetic tools to modulate the cell surface, a great plethora of functional groups can also be installed through metabolic glycoengineering. In these strategies, azide moieties (‐N3) have been the most explored and preferred groups to be inserted into the cell surface due to their high chemical stability in the in vivo environment. Relying on the initial installation of azide groups onto the cell surface through metabolic glycoengineering, researchers developed a double‐step strategy to promote cell–cell interactions and fabricate living cell assemblies.^[^ ^191^ ^]^ In this approach, a double click‐chemistry strategy was employed. Initially, installed azide groups were conjugated with heterobifunctional linkers, tetrazine (Tz)‐ dibenzocyclooctyne (DBCO) and *trans*‐cyclooctene (TCO)‐DBCO through strain‐promoted alkyne‐azide cycloaddition (SPAAC) reaction, installing the new functional moieties, Tz and TCO, in two separate cell lines. Both functional moieties promoted the covalent bond formation between co‐cultures of Jurkat T‐cells and A549/NIH‐3T3 cells, through a bioorthogonal click‐reaction, inverse‐electron‐demand Diels‐Alder (IEDDA), allowing the assembly between cell layers. These chemically interlinked layers exhibited a strong binding force and were able to support high shear stress levels. The binding force strongly depended on the amount of Tz and TCO groups available to promote multivalent linkages between cells. In the same study, homotypic clusters of Jurkat T‐cells were injected to investigate the in vivo stability of the cell–cell assemblies, revealing that most of the cell–cell pairs remained intact, proving their potential for in vivo applications. Owing to such stability, engineered cells arise as interesting building blocks that can be easily combined into larger assemblies, as well as be used for the development of cell‐based therapies. This technology was then improved to add reversibility to the cell assembly upon incorporation of a degradable disulfide bond between the heterobifunctional linker (DBCO‐SS‐TCO and DBCO‐SS‐Tz) (**Figure** **6**A).^[^ ^192^ ^]^ In the presence of non‐toxic glutathione (GSH) concentrations, disulfide bonds were successfully degraded, resulting in a quick detachment of cell assemblies, with ≈90% of dissociation. Moreover, metabolic glycoengineering can also be used to install photo‐responsive host‐guest moieties as intermediaries to attain a reversible mechanism of cell assembly.^[^ ^104^ ^]^ Cell surfaces were initially metabolically decorated with azide groups, that remained available for further bioorthogonal conjugation with an alkynyl group contained in a PEG‐modified β‐CD (Alkynyl‐PEG‐β‐CD) via copper(I)‐catalyzed azide‐alkyne cycloaddition (CuAAC) (Figure 6B). Relying on the host‐guest recognition between β‐CD, displayed at the cell surface, and azobenzene molecules, presented in a homobifunctional linker, cell–cell interactions were established through supramolecular binding, forming cell clusters. Due to the gap between the binding affinities of *trans*‐ and *cis*‐azobenzene with β‐CD and the possibility to reversibly interconvert the two isomers upon light irradiation, researchers were able to freely control the supramolecular cell‐binding and obtain spatiotemporal control over the cell–cell reversible interactions of the resultant homotypic assemblies (Figure 6B). Alongside, this system was endowed with targeting properties by exploring an azobenzene‐aptamer conjugate, maintaining the azobenzene as a photo‐switchable guest component and an aptamer for targeting MUC‐1 protein expressed on MCF‐7 breast cancer cells. By adding this target recognition property, reversible cell–cell interactions were successfully obtained within the co‐culture, allowing the fabrication of heterotypic living architectures. Upon UV irradiation such architectures disassemble and could then assemble once again in the presence of more azobenzene‐aptamer conjugates. The same supramolecular binding mechanism was also installed onto the cell surface through other approaches, such as hydrophobic insertion, namely by replacing the alkynyl group with a modified phospholipid (i.e., 1,2‐distearoyl‐*sn*‐glycero‐3‐phosphoethanolamine (DSPE)), allowing the resultant amphiphilic conjugate to be directly and rapidly incorporated into the cell membrane.^[^ ^193^ ^]^

The chemical decoration of the cell surface using ssDNAs can also be obtained through the metabolic installation of chemical moieties, improving the specificity of DNA insertion. In an elegant study, short oligonucleotide sequences were installed onto the cell surface through bioorthogonal Staudinger ligation or copper‐free click‐chemistry between modified ssDNA and azido‐glycoengineered Jurkat T‐cells.^[^ ^194^ ^]^ Hybridization of complementary sequences directed the formation of specific cell–cell interactions and the subsequent formation of 3D multicellular aggregates supported by DNA duplexes (Figure 6C). The technology is highly versatile and depends on DNA sequence complexity, insertion density, and cell concentration. Tuning these parameters opens new ways to modulate the cell assembly process. Interestingly, the disassembly can be triggered via controlled melting or degradation of duplex DNA linkages, deconstructing the structure into its initial building blocks.

Aiming to surpass monovalent modifications of the cell surface for the fabrication of microscale tissue‐like structures, researchers have recently developed an in situ branch‐like polyvalent DNA structure arising from a DNA initiator (DI) through chemical conjugation of a DBCO‐functionalized DI with a metabolically installed azide chemical group (Figure 6D).^[^ ^195^ ^]^ The DNA molecules were then amplified with DNA hairpins in situ through hybridization chain reaction prompting the self‐assembly of a branch‐like polyvalent DNA structure, containing multiple molecular recognition sites that are available to establish a selective cell–cell connection through hybridization between complementary DNA strands (Figure 6D). This technology led to the generation of robust cellular structures with improved cell–cell recognition. Importantly, this approach enables the establishment of multiple interactions with a low degree of membrane modification without significantly disturbing surface composition and functionality. By resorting to such building blocks, more robust living architectures can be fabricated when compared, for example, to monovalent DNA‐based interactions.

### 3.2 Programmable Quasi All‐Cellular Assemblies

Besides cell–cell interactions, in vivo tissue development is also synergistically governed by cell–matrix interactions that support numerous cellular and tissue functions through the coordination of dynamic biochemical and biomechanical cues.^[^ ^26^ ^]^ Recapitulating these important characteristics is critical for developing well‐organized tissue biomimetic architectures. In this context, natural and synthetic biomaterials have been exploited as extracellular matrix (ECM) analogs to provide a supportive microenvironment and promote biological function within living assemblies.^[^ ^38^ ^]^ To mitigate the relatively poor cellularization of conventional cell‐laden materials (i.e., low cell density to biomaterial ratio), new platforms are being developed through a synergistic combination of biomaterials and cell surface engineering methods. In such approaches, the required biomaterial fraction is minimal, and the cellular building blocks play an active role in the assembly process, yielding quasi all‐cellular assemblies that mostly exhibit cell‐governed dynamic biofunctionalities. In this context, the assembly process has been driven by the conjugation of surface‐engineered cells with different types of biomaterials, including, but not limited to polymers or polymeric nano/microparticles, proteins, etc. In these platforms, cells act as important structural elements that can selectively and actively modulate the formation of the resulting biomaterial‐mediated cell assemblies.

**Figure 7:**
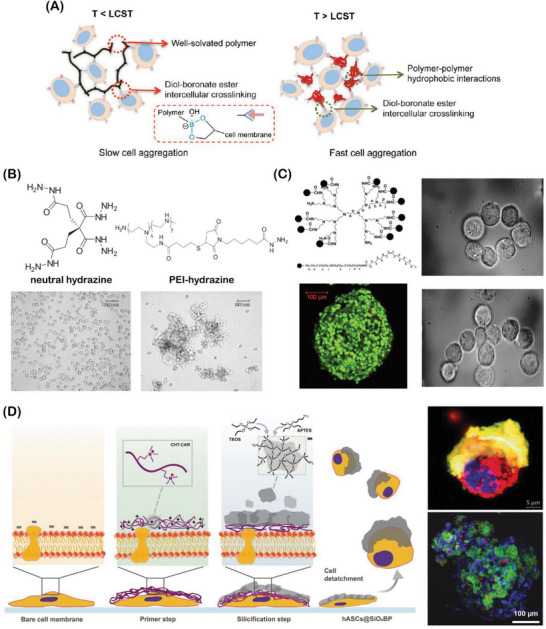
Assembly of cell‐rich polymeric materials via covalent, electrostatic, and hydrophobic cell surface functionalization. A) Schematic of cell surface modification with boronic acid copolymers to trigger an accelerated cell aggregation via covalent boronate‐ester bonds and polymer‐polymer hydrophobic interactions. Reproduced with permission.^[^ ^196^ ^]^ Copyright 2016, American Chemical Society. B) Electrostatic‐assisted cell aggregation via covalent modification of aldehyde groups from HepG2 cells population with hydrazine functionalized PEI polymeric chains. Reproduced with permission.^[^ ^68^ ^]^ Copyright 2007, Elsevier. C) Establishment of multicellular living assemblies mediated by electrostatic‐assisted hydrophobic insertion of dendrimeric intercellular linkers into C3A cell surface. CLSM micrographs of engineered assemblies after 7 days of culture. Scale bar: 100 µm. Fabrication of structures with defined shapes and patterns. Reproduced with permission.^[^ ^197^ ^]^ Copyright 2010, Elsevier. D) Schematic of silicification process promoted on top of electrostatically (partial) coated hASCs. CLSM micrograph of coated hASCs (upper) and widefield fluorescence micrograph showing larger cell aggregates (lower). Scale bars: 5 µm and 100 µm, respectively. Reproduced with permission.^[^ ^61^ ^]^ Copyright 2021, Wiley.

In this context, the installation of two copolymers into the cell surface, through direct chemical covalent modification resulted in a rapid, effective, and reversible cell aggregation.^[^ ^52^ ^]^ In this approach, a copolymer of *N*‐vinylpyrrolidone and 3‐(acrylamido)phenylboronic acid (APBA) was prepared and conjugated to *cis*‐diol groups on sialic acids through the formation of dynamic boronate‐ester bonds. Due to the dynamics of boronate‐ester bonds, cell assemblies were disassembled by introducing glucose into the system, where concentrations above 0.01 mm resulted in aggregate' disruption. In addition, the second copolymer aggregation system relied on the lower critical solution temperature (LCST) of the copolymer composed of di(ethylene glycol) methyl ether methacrylate and NHS methacrylate, which was covalently installed in the cell surface through the formation of amide bonds with surface primary amines. At temperatures above the polymers’ LCST, cellular assemblies were obtained by polymer‐polymer hydrophobic interactions. The resulting cell assembly proved to be completely reversible upon perturbation of the hydrophobic interactions by applying a temperature lower than LCST. The resulting cell–biomaterial platforms showed the potential of using polymer‐functionalized cells as building blocks to create self‐supportable, quasi all‐cellular assemblies with rapid and dynamic cell aggregation. This approach unveils interesting opportunities for diverse applications in tissue engineering, particularly the use of such quasi all‐cellular formulations as inks to be processed by 3D/4D bioprinting. In a follow‐up study, these copolymer cell‐aggregation mechanisms were combined into a single system promoting an accelerated and robust aggregation of highly responsive cellular assemblies and spheroids comprised of either cancer or cardiac cells.^[^ ^196^ ^]^ For this, researchers explored a copolymer comprised of a thermoresponsive polymer, poly(N‐isopropylacrylamide) (PNIPAAm), and APBA, that could be successfully attached to the *cis*‐diol units of the cell surface through boronate‐ester dynamic bonds (**Figure** **7**A). Owing to the dual properties of the copolymer, the system showed accelerated kinetics in cell aggregation via diol‐boronate intercellular crosslinking and polymer‐polymer hydrophobic interactions above LCST, these could simultaneously take place to drive the cell assembly process. Well‐defined spheroids were rapidly obtained at suitable operating temperatures and using rather low polymer concentrations (i.e., 25 µg mL^−1^). The developed quasi all‐cellular assemblies exhibited a controllable and stimuli‐responsive nature, whose aggregation properties and reversibility can be precisely tuned by controlling free glucose concentration and temperature. Leveraging this, the spheroid formation process was accelerated, further opening possibilities for their use as advanced building blocks for fabricating higher‐order architectures through biofabrication technologies (e.g., Kenzan method and aspiration‐based 3D bioprinting). Moreover, due to their increased robustness, such spheroids may be easier to handle, when compared to their conventionally generated counterparts.

Apart from these examples, other cell–biomaterial synergies can be explored for producing quasi all‐cellular assemblies. Driven by the negative charge of the cell membrane, cationic biomaterials can be installed through electrostatic binding, thus providing a polymeric interface for the fabrication of cell assemblies. However, electrostatic interactions are generally sub‐optimal for promoting the formation of stable cellular assemblies due to their relatively low stability in complex biological environments.^[^ ^69^ ^]^ Considering this, electrostatic binding is commonly employed in conjugation with other methods to improve efficacy and promote a higher aggregation stability. For example, fabrication of quasi all‐cellular assemblies was achieved by installing PEI functionalized with hydrazine moieties, into aldehyde moieties displayed at the cell surface via DCC.^[^ ^68^ ^]^ The incorporated inter‐cellular linker allowed a rapid and effective establishment of 3D living assemblies via covalent and electrostatic synergetic cooperation (Figure 7B). Despite the transient nature of the polymer linker, the multicellular assembly showed normal proliferation and was able to maintain the 3D morphology during 7 days in culture. Importantly, the cationic nature of PEI and resultant electrostatic interactions played a critical role in driving HepG2 cell aggregation into a spheroid‐like structure, by allowing the concentration of the functional moieties close to the cell surface, leading to larger cell–biomaterial assemblies with improved stability (Figure 7B). Similarly, a one‐step platform to promote the fabrication of multicellular structures was also reported by combining hydrophobic and electrostatic interactions, thus eliminating the pre‐conditioning chemical step to install the chemical moieties.^[^ ^197^ ^]^ In this platform, C3A cells were conjugated with a polymeric linker comprised of oleyl‐PEG conjugated to a 16 arm‐polypropylenimine hexadecaamine dendrimer (Figure 7C). Due to the synergistic action of dendrimer positive charge and hydrophobic oleyl moieties, the multivalent dendrimeric inter‐cellular linker was able to be successfully attached to the cell membrane via simultaneous hydrophobic insertion and electrostatic binding, promoting rapid and effective cell aggregation upon centrifugation. Moreover, these building blocks were successfully applied to fabricate multicellular structures with defined shapes and patterns using optical tweezers to control and manipulate cell position, revealing to be interesting materials for the assembly of more complex tissue‐like architectures with control over spatial arrangements (Figure 7C). Alternatively, electrostatic binding has also been explored to drive silicification processes, generating a protective and supportive partial silica shell on the cell surface.^[^ ^61^ ^]^ In this approach, hard silica backpacks were successfully installed on human adipose‐derived MSCs (hASCs) surface by following a double‐step protocol. To bypass potential cytotoxic effects associated with highly positive polycations, a carnitine‐modified chitosan (CHT‐CAR) was used as the first polycationic layer that interacted with the negatively charged cell surface. Following the addition of tetraethyl orthosilicate and (3‐aminopropyl)triethoxysilane, a silica structure was formed on top of the polycationic layer and partially covered the cell surface (Figure 7D). This innovative platform provided cells with mechanical support and anchorage points, allowing them to spread and establish larger aggregates. Such could, in turn, influence stem cell fate, by inducing differentiation toward bone lineages. Considering this, the incorporation of silica backpacks may provide a new class of engineered cell units that can be explored for bone tissue engineering or other cell therapies that benefit from stem cell delivery. Moreover, the mechanical resistance provided by the backpack may allow the processing of these building blocks under higher shear stresses, improving their handling via extrusion 3D bioprinting. Additionally, the backpack could be tailored with chemical cues, allowing it to modulate and control cell behavior and fate during and after the assembly process.

**Figure 8:**
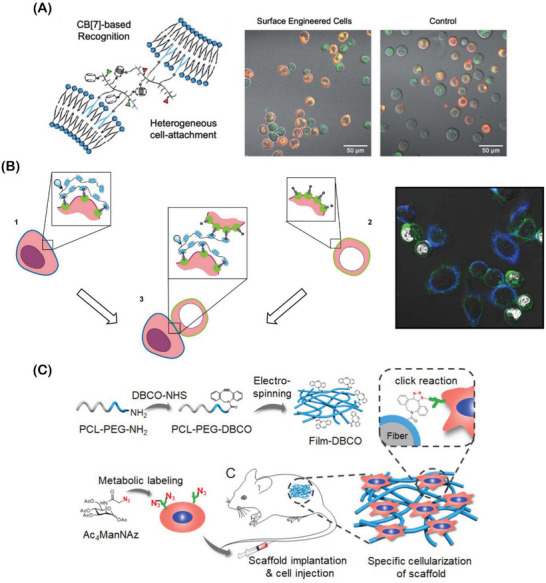
A) Illustration of selective supramolecular cell–biomaterial aggregates based on the hydrophobic insertion of multi‐functional polymeric chains bearing CB[7]/Ada host‐guest pairs. CLSM micrographs of heterogeneous attachment between dendritic and cancer cells. Scale bar: 50 µm. Reproduced with permission.^[^ ^105^ ^]^ Copyright 2022, Royal Society of Chemistry. B) Schematic illustration of a biomolecular recognition platform for cell functionalization and selective cell capture through the specific installation of Ac‐TZ14011‐Ada peptide targeted to the CXCR4 receptor, overexpressed in breast cancer cell's surface. CLSM micrograph of induced cellular interactions between surface‐engineered MDA‐MB‐231 cell populations and Cy3_1.5_CD_10_PIBMA_389_‐interfacing polymer via supramolecular Ada/β‐CD interactions. Reproduced under the terms of the CC‐BY Creative Commons Attribution 4.0 International License (http://creativecommons.org/licenses/by/4.0/).^[^ ^199^ ^]^ Copyright 2017, The Authors, Published by Springer Nature. C) Schematic of in vivo top‐down assembly of cell–biomaterial living platforms with selective cell binding properties based on click‐chemistry toolboxes. Cellular interactions are established between injected metabolic glycoengineered MDA‐MB‐231 breast cancer cell populations exhibiting azide (‐N3) functionalities and a subcutaneous implanted electrospun microfiber scaffold bearing complementary DBCO moieties. Reproduced with permission.^[^ ^200^ ^]^ Copyright 2020, Elsevier.

Additionally, the hydrophobic nature of the cell membrane by itself can act as a potential crosslinker to develop gel‐like networks using cells as an active building block to trigger the gelation process.^[^ ^198^ ^]^ Hydrophobically modified chitosan and alginate were successfully embedded in the cell membrane through hydrophobic anchoring of alkyl chains, triggering a rapid transition into a self‐supporting gel, without requiring additional crosslinking reagents. Moreover, due to the weaknesses of hydrophobic interactions, a disassembly process can be triggered by the addition of an α‐CD) competitor, reverting the cell‐gel state and disassembling the building blocks. Due to its shear‐thinning properties, this generic platform could be used as an injectable formulation for 3D/4D bioprinting applications. In line with this approach, a supramolecular cell–biomaterial platform was developed through the installation of norbornene polymeric chains functionalized with CB[7] and Ada (NB‐DSPE‐CB[7] and NB‐DSPE‐Ada, respectively), containing modified phospholipids (DSPE) as hydrophobic anchors.^[^ ^105^ ^]^ The formed agglomerates, relied on the intermediary recognition system between CB[7]/Ada host‐guest pair, that promoted a selective dendritic and cancer cells heterogeneous assembly (**Figure** **8**A). Relying on the specific biomolecular recognition between chemokine receptor 4 (CXCR4) and the peptide TZ14011, a similar platform was developed by employing a two‐step process to promote cell aggregation.^[^ ^199^ ^]^ For this, adamantane molecules linked to Ac‐TZ14011 peptide (Ac‐TZ14011‐Ada) were installed on the target cell surface through the specific binding between the peptide and CXCR4 surface receptor, exposing the guest molecule for a secondary supramolecular functionalization with multivalent β‐CD moieties displayed in poly(isobutylene‐*alt*‐maleic‐anhydride) (PIBMA) polymeric chains (Figure 8B). Supramolecular biomaterial‐based aggregation of targeted adamantane functionalized cells was promoted, allowing control over the recognition and reversibility of the assembly.

By exploring the tyrosine residues displayed on ECDs of certain proteins, the cell surface can be functionalized through an enzymatic‐mediated oxidative crosslinking between these naturally occurring phenolic moieties and a tyramine‐functionalized dextran.^[^ ^146^ ^]^ This technology, so termed DOCKING, allows the covalent tethering of cells to phenolic functionalized biomaterials, reducing the potential cytotoxicity effects of conventional cell‐surface adhesive biomaterials (i.e., cationic polymers, etc.). This strategy provides temporal control over the mechanotransducive properties of the assembly, allowing one to reprogram cell fate and study cell–material interactions at single‐cell resolution within 3D cell assemblies. Functional groups installed through metabolic glycoengineering can also be used to anchor cells and further anchor long polymeric chains for the fabrication of quasi all‐cellular assemblies with minimal fraction of biomaterials. Resorting to this technology, researchers supplied cells with methacryoyl‐modified *N*‐acetyl mannosamine residues to display methacryoyl groups in cell surface sialic acid residues.^[^ ^201^ ^]^ After this installation, polymeric chains were anchored to the cell surface through conjugation with terminal thiols contained in thiol‐modified PNIPAAm by a thiol‐ene reaction promoted by UV irradiation. Due to the thermoresponsive properties of PNIPAAm, cell aggregation could be reversibly manipulated by changing the temperature above or below its LCST, similar to previously discussed technologies. This approach shows potential for promoting the formation of complex aggregates with improved spatiotemporal resolution over the assembly process owing to the light‐dependency of the thiol‐ene reaction. Living bulk hydrogels were also developed by exploring metabolically labeled cells as active cross‐linking elements for the development of cell‐rich materials at the macro‐scale.^[^ ^202^ ^]^ In this elegant approach, azide groups were metabolically incorporated into the cell surface and conjugated with DBCO‐modified branched alginate by a bioorthogonal SPAAC reaction. This azide‐cell/biomaterial combination resulted in the formation of multifunctional living hydrogels with unique functionalities including self‐proliferation, self‐degradability, and selective cell adhesion. Moreover, this concept was used in vivo by using cells obtained from mouse tissues that were further processed to develop cell‐crosslinked hydrogels. Interestingly, these hydrogels were able to form in situ in multiple tissues, including lung, heart, muscle, and kidney, proving the potential of this generic approach for fabricating higher‐order quasi all‐cellular assemblies.

From a top‐down perspective, cell surface engineering could also be combined with conventional tissue engineering scaffolds to improve their cellularization levels, thus addressing the poor in vivo cellularization commonly found upon transplantation. Considering this, metabolically engineered cells, displaying azide groups on their surface, were combined with a subcutaneously implanted film of polycaprolactone‐PEG‐DBCO microfibers obtained through electrospinning (Figure 8C).^[^ ^200^ ^]^ The bioorthogonal conjugation between azide‐functionalized macrophages and DBCO moieties in the scaffold surface, led to an improved cell attachment and survival, with increased cell‐attachment selectivity and controlled in situ cellularization being observed following in vivo injection of free cells. Similar concepts have also been explored for the development of scaffolds with avidin moieties that could be recognized by specific biotinylated chondrocytes resulting in enhanced recruitment, thus boosting the in vivo regeneration process.^[^ ^203^ ^]^ These rapidly emerging approaches evidence the immense possibilities of exploring minimalistic biomaterial amounts as interfacing elements to promote the fabrication of programmable multicellular assemblies. Further advancements focusing on the use of ECM‐mimetic materials for these interfaces are expected.

## 4 Outlook, Challenges, and Potential Advancements

From a holistic perspective, cell surface engineering has provided innovative ways to convert cells into functional building blocks with programmable features that enable on‐demand or self‐governed interactions that can ultimately be leveraged to generate complex multicellular architectures with unique features and bioactivities. So far, by employing surface‐engineered living units, researchers have been able to recapitulate key aspects of in vivo tissue development including cellular self‐sorting, symmetry breaking, as well as dynamic and reversible cell–cell/cell–matrix interactions that are challenging to achieve in conventional tissue engineering approaches.

Despite the remarkable progress in cell surface engineering, several challenges and limitations still need to be addressed in the field. An important aspect to consider at the design stage is the biocompatibility of the modification techniques. Introducing foreign molecules or materials onto cell surfaces should not compromise cell viability or biofunctionality. Therefore, it is necessary to further optimize cell surface functionalization methods and carefully select the chemistry/biomaterials used to minimize any adverse effects. Furthermore, the scalability and reproducibility of cell surface engineering techniques are critical for their translation into realistic applications. Developing simplified, cost‐effective, and increasingly standardized approaches will facilitate the broader adoption of cell surface engineering in various fields. Another key challenge is to ensure the stability and longevity of the modified cells. The functional molecules attached to the cell surface may undergo degradation or detachment over time. Therefore, strategies to enhance the stability of cell surface modifications and improve their long‐term performance are crucial. Relatively extended functionalization lifetimes have already been achieved in hydrophobic insertion techniques with the use of engineered conjugates (i.e., DNA tetrahedron linked to cholesterol anchors).^[^ ^77^ ^]^ The installation of DNA is particularly valuable since DNA reaction circuits used to establish different assembly pathways can be precisely controlled by sequential allosteric activation.^[^ ^204^ ^]^ We envision that future developments with the combination of DNA nanotechnology and cell surface engineering will expand the possibilities to further modulate cellular interactions. Alternatively, the installation of synthetic polymers on the cell surface may also offer unconventional possibilities for designing cell‐rich assemblies with relatively extended functionalization lifetimes. Typical strategies for polymer functionalization involving “grafting to” approaches display, however, a relatively low grafting efficiency or require an excess of polymer to attain a suitable degree of functionalization.^[^ ^38^, ^205^ ^]^ Conversely, “grafting from” strategies can be further explored (i.e., surface‐initiated controlled radical polymerization) to increase grafting efficiency and functionalization lifetime while maintaining viability and cell surface bioactivity.^[^ ^63^, ^205^ ^]^ Such concept has been recently applied in mammalian cells by using photoinduced electron transfer‐reversible addition‐fragmentation chain‐transfer polymerization (PET‐RAFT), which provided an alternative approach for installing well‐defined and distributed polymeric chains on the cell surface.^[^ ^205^ ^]^

Adding to these improvements, exploring computational tools such as artificial intelligence/machine learning (AI/ML) approaches to cell surface engineering is also envisioned to provide alternatives to better predict cellular interactions and self‐sorting events. This can allow the identification of possible triggers for generating specific cellular arrangements in more complex multicellular living assemblies across time.^[^ ^206^, ^207^ ^]^ This approach has been recently applied in cells engineered via the synNotch technology, where in silico modeling was applied to identify the parameters required to attain a self‐assembled symmetrical four‐layered spheroid, solely based on cadherin‐mediated cell adhesion.^[^ ^206^ ^]^ Further exploring in silico‐assisted design to identify less intuitive setups could drive non‐conventional cell organizations and optimized morphogenesis processes.^[^ ^27^, ^208^ ^]^ For example, we envision that identifying key parameters for spatiotemporal control of cellular organization could help mitigate common patterning and growth variabilities observed in organoid morphogenesis.^[^ ^209^ ^]^ Merging such modeling tools with multi‐omics analysis will further contribute to a better understanding of the biological features of such complex organotypic assemblies.

Considering the increased interest in such lab‐grown tissues, there is also a need to promote cell assembly at the macro‐scale in a faster and more reproducible manner.^[^ ^21^ ^]^ Surface‐engineered cells have already been used for macro‐scale muscle repair and for promoting a faster generation of functional liver or cardiac tissues when compared to assemblies generated via native cellular adhesions of pristine cells.^[^ ^32^, ^178^, ^186^ ^]^ Advances in biofabrication technologies (i.e., digital light processing, volumetric‐, aspiration‐ or microfluidic‐based bioprinting) may further enable researchers to process functionalized cells into macro‐scale living materials with higher spatiotemporal control over tissue organization. This could unlock the so‐desired fabrication of cell‐dense tissues in a more reproducible and controlled mode.^[^ ^210^, ^211^, ^212^ ^]^ The convergence of biofabrication and cell surface engineering is still in its infancy, and numerous opportunities exist for generating organotypic macro‐scaled assemblies of various human tissues in the future.^[^ ^213^ ^]^

In conclusion, continuous advancements in the cell surface engineering field are expected to pave the way for developing increasingly biofunctional multicellular therapeutics and living materials that will find broad applications in healthcare and biotechnology.

## Conflict of Interest

The authors declare no conflict of interest.
